# Expanding the CarD interaction network: CrsL is a novel transcription regulator in actinobacteria

**DOI:** 10.1093/nar/gkaf1342

**Published:** 2025-12-17

**Authors:** Mahmoud Shoman, Martin Černý, Jitka Jirát Matějčková, Marek Schwarz, Nabajyoti Borah, Viola Vaňková Hausnerová, Silvia Neva, Michaela Šiková, Hana Šanderová, Petr Halada, Martin Hubálek, Věra Dvořáková, Martin Převorovský, Jana Holubová, Ondřej Staněk, Libor Krásný, Lukáš Žídek, Jarmila Hnilicová

**Affiliations:** Laboratory of Regulatory RNAs, Faculty of Science, Charles University, Prague 12844, Czech Republic; Laboratory of Microbial Genetics and Gene Expression, Institute of Microbiology of the Czech Academy of Sciences, Prague 14220, Czech Republic; Central European Institute of Technology (CEITEC), Masaryk University, Brno 62500, Czech Republic; National Centre for Biomolecular Research, Faculty of Science, Masaryk University, Brno 62500, Czech Republic; Laboratory of Regulatory RNAs, Faculty of Science, Charles University, Prague 12844, Czech Republic; Laboratory of Bioinformatics, Institute of Microbiology of the Czech Academy of Sciences, Prague 14220, Czech Republic; Laboratory of Microbial Genetics and Gene Expression, Institute of Microbiology of the Czech Academy of Sciences, Prague 14220, Czech Republic; Laboratory of Regulatory RNAs, Faculty of Science, Charles University, Prague 12844, Czech Republic; Laboratory of Regulatory RNAs, Faculty of Science, Charles University, Prague 12844, Czech Republic; Laboratory of Microbial Genetics and Gene Expression, Institute of Microbiology of the Czech Academy of Sciences, Prague 14220, Czech Republic; Laboratory of Microbial Genetics and Gene Expression, Institute of Microbiology of the Czech Academy of Sciences, Prague 14220, Czech Republic; Laboratory of Structural Biology and Cell Signaling, BIOCEV, Institute of Microbiology of the Czech Academy of Sciences, Vestec 25250, Czech Republic; Institute of Organic Chemistry and Biochemistry of the Czech Academy of Sciences, Prague 16000, Czech Republic; Military Health Institute, Military Medical Agency, Prague 16200, Czech Republic; National Institute of Public Health, Prague 10000, Czech Republic; Department of Cell Biology, Faculty of Science, Charles University, Prague 12800, Czech Republic; Laboratory of Molecular Biology of Bacterial Pathogen, Institute of Microbiology of the Czech Academy of Sciences, Prague 14220, Czech Republic; Laboratory of Molecular Biology of Bacterial Pathogen, Institute of Microbiology of the Czech Academy of Sciences, Prague 14220, Czech Republic; Laboratory of Microbial Genetics and Gene Expression, Institute of Microbiology of the Czech Academy of Sciences, Prague 14220, Czech Republic; Central European Institute of Technology (CEITEC), Masaryk University, Brno 62500, Czech Republic; National Centre for Biomolecular Research, Faculty of Science, Masaryk University, Brno 62500, Czech Republic; Laboratory of Regulatory RNAs, Faculty of Science, Charles University, Prague 12844, Czech Republic; Laboratory of Microbial Genetics and Gene Expression, Institute of Microbiology of the Czech Academy of Sciences, Prague 14220, Czech Republic

## Abstract

Bacterial transcription regulation is critical for adaptation and survival. CarD is an essential transcription factor in mycobacteria involved in the regulation of gene expression. We searched for CarD interaction partners in *Mycobacterium smegmatis* and identified a novel uncharacterized protein, named CrsL (*MSMEG_5890*). CrsL is a 5.7 kDa protein shown by NMR to be intrinsically disordered. CrsL homologs are present in actinobacteria, including pathogenic species such as *Mycobacterium tuberculosis*. CrsL interacts directly with CarD, adopting an ordered structure in the complex, and also binds RNAP, controlling CarD–RNAP association. ChIP-seq showed that CrsL associates with the promoters of actively transcribed genes and ∼75% of these regions are also associated with CarD. RNA-seq revealed ∼50% and ∼66% overlap in differentially expressed genes between CrsL and CarD knockdowns during the exponential and stationary phases, respectively. Among CrsL-regulated genes are DesA desaturase (*MSMEG_5773*) and DEAD/DEAH-box RNA helicase *MSMEG_1930*, which contribute to cold stress adaptation. CrsL supports the growth of *M. smegmatis* at elevated temperature but limits growth in cold environments. In summary, these findings identify CrsL as a novel, conserved CarD-interacting protein playing a key role in mycobacterial stress responses by modulating CarD function.

## Graphical Abstract

**Graphical Abstract:**
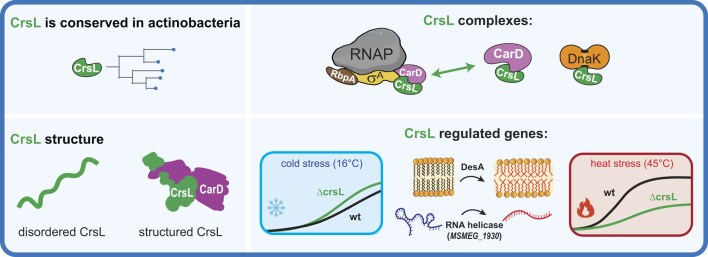

## Introduction

Mycobacteria belong to the actinobacteria and include slow-growing species such as *Mycobacterium tuberculosis*, which is responsible for tuberculosis, as well as many rapidly growing nontuberculous species that can cause various infections. Certain nontuberculous mycobacteria are capable of growth in environments with a wide range of temperatures, including the water circuits of heater-cooler units—medical devices that control the patient’s body temperature during open-heart surgery. *Mycobacterium chimaera* was reported to infect patients during cardiac surgeries when they are exposed to contaminated aerosols from these units [1, 2]. Like other bacteria, mycobacteria rely on transcriptional regulation to survive varying temperatures and environmental challenges. In this study, we examined transcriptional regulation in *Mycobacterium smegmatis*, a well-established model for investigating these regulatory mechanisms in mycobacteria.

Bacterial transcription involves the synthesis of RNA from a DNA template, a process mediated by a single type of DNA-dependent RNA polymerase (RNAP) [3]. The RNAP core consists of several subunits (α2ββ′ω) [4]. These subunits associate with a sigma (σ) factor to form the RNAP holoenzyme, that can recognize promoter sequences and initiate transcription [5–7]. All bacteria have one primary σ factor [7, 8]. The primary σ factor in mycobacteria is σ^A^ and is essential for bacterial growth [9, 10]. In addition to σ^A^, the mycobacterial alternative σ factor σ^B^ recognizes similar promoter sequences to σ^A^, and consequently, the regulons of these σ factors partly overlap [11–14]. Furthermore, σ^B^ regulates stress-responsive genes [15–17].

The mycobacterial transcription machinery requires the additional transcription factors, RbpA and CarD, which are not present in *Escherichia coli* [18, 19]. These factors are essential global regulators in both *M. smegmatis* and *M. tuberculosis* [20–24].

RbpA consists of four domains and binds to RNAP containing either σ^A^ or σ^B^, but not the other alternative σ factors [20, 25–27]. It assists in promoter unwinding and the formation of the catalytically active open complex [11, 23]. RbpA was proposed to modify the structure of the RNAP core, increasing the competitiveness of the σ^A^ over the alternative σ factors [27]. Additionally, RbpA has been suggested to play a role in the release of rifampicin from RNAP and is also involved in the stress response [22, 27–30].

CarD consists of two domains. The CarD N-terminal domain has a conserved structure and interacts with RNAP (RNAP interaction domain, RID), while the C-terminal domain (CTD) binds to DNA (DNA-binding domain, DBD) [31]. CarD affects formation/stability of the open complex in a promoter-dependent manner [32]. It acts as a global transcription regulator [31–35] and regulates many genes including ribosomal RNAs encoding genes [33, 36]. Mycobacterial growth is altered when CarD function is disrupted by mutations targeting the RID, the DBD, or a conserved tryptophan residue (Trp85) [36]. Furthermore, weakening the mycobacterial RNAP–CarD interaction results in cells being more sensitive to stress conditions, including oxidative stress, DNA damage, and the effects of certain antibiotics [37, 38]. The level of CarD decreases during stationary phase and starvation. This decrease is mediated by *carD* antisense RNA and Clp protease [38].

Apart from CarD and RbpA, mycobacteria contain a unique ∼300 nt long Ms1 RNA [39]. Ms1 RNA is highly expressed during the stationary phase of *M. smegmatis* growth, interacting directly with the RNAP core without any σ factor to regulate the RNAP level in *M. smegmatis* [40, 41]. Ms1 has homologs in various actinobacterial species [42], including MTS2823 RNA, which is highly expressed in *M. tuberculosis* [43] and interacts with RNAP [44].

The aim of this study was to expand our knowledge of the mycobacterial transcription machinery by searching for CarD interaction partners in *M. smegmatis*. We identified a novel CarD interaction partner, CrsL. First, we validated the CrsL–CarD interaction and demonstrated that both proteins can associate with RNAP. CrsL tightly associates with CarD and influences its interaction with RNAP. We characterized the structure of CrsL, defined its binding sites across the chromosome and determined the effects of CrsL depletion on the transcriptome and the bacterial growth. CrsL regulates genes involved in temperature adaptation and promotes mycobacterial growth at elevated temperatures. Overall, our data suggest that CrsL is a novel mycobacterial transcription regulator.

## Materials and methods

### Construction of the bacterial strains

A detailed description of all bacterial strains, their construction, and the oligonucleotide sequences is provided in the Supplementary Data.

### Bacterial growth conditions

*M. smegmatis* mc^2^ 155 strains were grown on Middlebrook 7H10 (Difco) for 2–3 days at 37°C. When required, the media was supplemented with the following antibiotics: kanamycin (20–25 μg/ml) or hygromycin (50 μg/ml). The strains were inoculated into an overnight culture of Middlebrook 7H9 media (Difco), supplemented with 0.2% glycerol and 0.05% Tween 80 at 37°C. The overnight cultures were then diluted to OD_600_ 0.1 and allowed to grow to the exponential phase (OD_600_ ∼0.5; 6 h of growth) or the early stationary phase (OD_600_ ∼2.5–3; 24 h of growth).

FLAG-tagged and CRISPR knockdown strains: The strains were inoculated to OD_600_ 0.1. For exponential phase samples (6 h growth, OD_600_ ∼0.5), anhydrotetracycline (ATc) was added 3 h after inoculation in different concentrations: 100 ng/ml (for the CRISPR strains), 10 ng/ml (for ChIP-seq and IP), 25 ng/ml (for overexpression), or 1 ng/ml (for CarD–FLAG optimization), and cells harvested after an additional 3 h. For the stationary phase (24 h growth, OD_600_ ∼2.5–3), ATc was added 8 h after inoculation, and the cells harvested after 16 h later. For CarD–FLAG optimization, the ATc (1 ng/ml) was added after 21 h and the cells harvested after 3 h later.

For the growth curves at low or elevated temperatures, overnight cultures of the wt, ∆*crsL*, and ∆*crsL + crsL* strains were grown at the optimal temperature (37°C). After dilution to OD_600_ ∼0.1, the cells were grown at 37°C, 45°C, or 16°C. Initially, overnight cultures of *nc*WT and *crsL* knockdown strains were grown at the optimal temperature (37°C) with ATc (100 ng/ml) for ~16 h to deplete CrsL. These cultures were then diluted to OD_600_ ∼0.1 in 7H9 media with ATc (100 ng/ml). Six hours after inoculation, the temperature was raised to 45°C, after which the cells were cultivated for ∼35 h. The cells were grown in a Biosan RTS-8 Multi-Channel Bioreactor, and the OD_600_ was measured throughout the growth period.

### Immunoprecipitation (FLAG pulldown)

60–120 ml of *M. smegmatis* cells in the exponential and stationary phases were pelleted, washed in lysis buffer (20 mM Tris–HCl, pH 7.9, 150 mM KCl, and 1 mM MgCl_2_) and pelleted again. The cells were then stored at –80°C. The pellets were then resuspended in 3 ml of Lysis buffer supplemented with phenylmethylsulfonyl fluoride (PMSF) and Protease inhibitor cocktail [20 mM Tris–HCl pH 7.9, 150 mM KCl, 1 mM MgCl_2_, 1 mM dithiothreitol (DTT), 0.5 mM PMSF, Protease Inhibitor Cocktail Set III protease inhibitors (Calbiochem)], sonicated 15 × 10 s with 1 min pauses on ice and centrifuged at 8960 × *g* for 15 min at 4°C. An equal amount of cell lysates (2–4 mg of proteins) from the FLAG-tagged strains was incubated for 16–18 h overnight at 4°C with 10–25 μl of M2 anti-FLAG resin (Sigma–Aldrich). The protein complexes captured on the agarose gel beads were then washed 4 × with 0.5 ml of lysis buffer. The FLAG-tagged proteins were eluted by 60 μl of 3 × FLAG Peptide (Sigma F4799), diluted in Tris-buffer saline (TBS: 50 mM Tris–HCl pH 7.5, 150 mM NaCl) to a final concentration of 150 ng/ml. Alternatively, the FLAG-tagged proteins were released by boiling the beads in 4× SDS sample buffer for 5 min at 95°C. The proteins captured in the complexes were resolved on SDS–PAGE gels (Nu-PAGE, 4–12% Bis–Tris precast gels, Invitrogen) and stained using SimplyBlue SafeStain (Invitrogen) or stained with silver (Pierce Silver Stain Kit for Mass Spectrometry) or a SilverQuest Silver Staining Kit (Thermo Fisher Scientific), and/or analyzed by western blotting. The identity of the protein bands was determined by MALDI-FTICR mass spectrometry, as previously described [45].

### CrsL-His protein purification for *in vitro* assay and antibody production

The *E. coli* strains used for protein purification, CarD-NT (*LK3209*), CrsL-His (*LK3499*), and DnaK-His (*JH185*), were inoculated into overnight cultures in LB medium at 37°C. These cultures were then diluted to OD_600_ 0.03 into the LB medium with ampicillin (100 μg/ml) and shaken at 120 rpm at 37°C for ∼3 h (until OD_600_ ∼0.6). Expression was then induced with 0.8 mM IPTG (Isopropyl β-D-thiogalactopyranoside). The temperature was then lowered to room temperature (∼20°C) and the cultures were shaken for an additional 3 h. The cultures were cooled and centrifuged using a Beckman Coulter JA-10 rotor at 4°C, 6000 × *g* for 10 min. The pellets were resuspended in 1× T-buffer (300 mM NaCl, 50 mM Tris–HCl pH 7.5, and 5% glycerol), then centrifuged 5400 × *g* for 10 min, and the pellets were stored in −80°C. For DnaK purification, 1× P-buffer (300 mM NaCl, 50 mM Na_2_HPO_4_, 5% glycerol, 3 mM β-mercaptoethanol, and 0.1 mM PMSF) was used instead of 1× T-buffer throughout the procedure. The pellets were sonicated (Sonopuls HD3100, Bandelin [Germany]; 50% amplitude, 15 × 10 s pulse, 1 min pause on ice) in 1× T-buffer (supplemented with 3 mM β-mercaptoethanol). The samples were centrifuged at 27 000 × *g*, 4°C (Beckman Coulter, JA 25–50 rotor) for 10 min, and the cell lysates were incubated with 1 ml of prewashed Ni-NTA Agarose beads (QIAGEN) shaking at 4°C for 1.5 h. The samples were centrifuged at 2000 rpm, 4°C for 5 min, and Ni-NTA beads were resuspended in 10 ml of 1× T-buffer and centrifuged again at 2000 rpm, 4°C for 5 min. The Ni-NTA beads were resuspended in 10 ml of 1× T-Buffer (containing 30 mM imidazole) and centrifuged again at 2000 rpm, 4°C for 5 min. The Ni-NTA beads were then resuspended in 500 μl of 1× T-Buffer (400 mM imidazole), and serial fractions of the eluted proteins were collected. Their concentrations and purity were then measured using a Bradford assay and on SDS–PAGE, respectively. Selected fractions containing the DnaK protein were dialyzed against a storage buffer (50 mM Tris–HCl pH 8, 100 mM NaCl, 50% glycerol, and 3 mM β-mercaptoethanol) and stored at −20°C.

The selected fractions containing the CrsL–His protein with the highest concentration and the highest purity were pooled and dialyzed against CrsL dialysis buffer (50 mM Tris–HCl pH 8, 100 mM NaCl, 3 mM β-mercaptoethanol, and 5% glycerol) in Slide-A-Lyzer Dialysis Cassette 2 000 MWCO (Thermo Fisher Scientific). The CrsL–His protein was then further purified using the ÄKTA pure^™^ chromatography system (Superdex 75 column). The protein fractions of higher purity were pooled and dialyzed against a storage buffer (50 mM Tris–HCl, pH 8, 100 mM NaCl, 50% glycerol, and 3 mM β-mercaptoethanol) and stored at −20°C. The CrsL-His protein was then used for mice immunization and anti-CrsL antibody production, as described in the following section.

The purification protocol for the CarD–NT protein was similar to that described above, with the inclusion of TEV protease cleavage, as previously described [46, 47].

### Animal experiments and immunizations

All animal experiments were approved by the Animal Welfare Committee of the Institute of Molecular Genetics of the Czech Academy of Sciences, v. v. i., in Prague, Czech Republic. The handling of animals was performed according to the Guidelines for the Care and Use of Laboratory Animals, the Act of the Czech National Assembly, Collection of Laws No. 246/1992. Permission No. 19/2020 was issued by the Animal Welfare Committee of the Institute of Molecular Genetics of the Czech Academy of Sciences in Prague.

Five-week old female BALB/cByJ mice (Charles River, France) were immunized via intraperitoneal injection with CrsL protein (20 μg in 200 μl) adjuvanted with aluminum hydroxide (Alum, SevaPharma, Czech Republic). The mice received three doses of the protein at 2-week interval. One week after the third immunization, blood was collected from anesthetized animals (i.p. injection of 80 mg/kg ketamine and 8 mg/kg xylazine) using the retroorbital puncture method. The sera were recovered from the supernatant after centrifugation of clogged blood at 5000 × *g* for 10 min at 8°C and stored at –20°C. The specificity of the antibody was checked using 1 μg of *M. smegmatis* cell lysate with 1:5000 antibody dilution.

### Western blotting and FAR-western blotting

The samples were resolved on SDS–PAGE gels and detected by western blotting using anti-RNAP β subunit antibody [clone 8RB13] (BioLegend) or anti-σ^70^ antibody [clone 2G10] (BioLegend), anti-CarD antibody, anti-RbpA antibody, anti-CrsL antibody, anti-GroEL [5177] antibody (Santa Cruz Biotechnology), as well as a HRP-labeled anti-mouse IgG antibody (Sigma–Aldrich). The blots were incubated with SuperSignal West Pico PLUS Chemiluminescent substrate (Thermo Fisher Scientific), after which the signals were detected either on film or using G-box (Syngene) with different exposure times.

FAR-western blotting was performed according to the previously established protocol [48]. Briefly, the BSA, CarD–NT, and CrsL–His proteins were loaded in duplicate, resolved on SDS–PAGE gels and blotted onto Amersham Protran Nitrocellulose Membrane (Sigma–Aldrich). The membranes were then incubated with decreasing concentrations of guanidine–HCl (Thermo Fisher Scientific) for protein denaturation and renaturation. The membranes were blocked with 5% milk and washed with PBST buffer. The membranes were cut into two parts. The first part (negative control) was incubated directly with an anti-CarD antibody. The second part was first incubated with purified CarD protein (20 μg in 10 ml) overnight to allow CrsL–CarD complex formation. It was then incubated with the anti-CarD antibody. The membranes were then washed with PBST and incubated with HRP-labeled anti-mouse secondary antibody (Sigma–Aldrich), after which the signals were detected.

### Native gel electrophoresis

Binding reactions were performed in 8 μl of 1× STB buffer (50 mM Tris–HCl pH 8.0, 5 mM Mg(C_2_H_3_O_2_)_2_, 100 μM DTT, 50 mM KCl, and 50 μg/ml BSA) containing *in vitro* purified CrsL, CarD, and DnaK proteins. The proteins were reconstituted at 37°C 15 min and 8 μl of 2× Loading dye (62.5 mM Tris–HCl, pH 6.8, 25% glycerol, and 1% Bromophenol Blue) was added. The samples were then loaded on 8% native gel [40% acrylamide–Solution 19:1 (AppliChem), 0.375 M Tris–HCl (pH 8.8), 10% (w/v) ammonium persulfate, TEMED]. Electrophoresis was performed at 4°C, 150 V (15 min pre-run followed by a 3 h run) using a running buffer (25 mM Tris and 192 mM glycine). The gels were subsequently stained with SimplyBlue (Invitrogen) for protein visualization and the identity of the protein bands confirmed by mass spectrometry.

### ChIP-seq

CrsL-FLAG ChIP-seq experiments were performed in parallel with HelD, RbpA, and CarD ChIP-seq [46]. Briefly, 2 mg of protein cell lysates was incubated with 20 μl of M2 anti-FLAG resin (Sigma–Aldrich). The captured complexes were then washed twice with RIPA buffer (150 mM NaCl, 1% Triton X-100, 0.5% deoxycholate, 0.1% SDS, 50 mM Tris–HCl pH 8.0, and 0.5 mM EDTA), four times with LiCl buffer (100 mM Tris–HCl, pH 8.5, 500 mM LiCl, 1% Triton X-100, and 1% deoxycholate), two times with RIPA, and twice with TE buffer (10 mM Tris–HCl, pH 8.0, and 1 mM EDTA). The protein–DNA complexes were then eluted with an elution buffer (50 mM Tris–HCl pH 8, 0.66 mM EDTA, and 1% SDS) for 10 min at 65°C, decrosslinked in the presence of 200 mM NaCl for 5 h at 65°C and treated with 100 µg/ml RNase A for 1 h at 37°C, and 400 µg/ml proteinase K for 30 min at 45°C. The DNA was then purified with the QIAGEN PCR purification kit and eluted with 100 μl of Elution Buffer. 40 µl of immunoprecipitated DNA sample or 10 ng of the DNA input were used for library construction, according to the NEXTFLEX ChIP-Seq Kit manual, including the Size-Selection Cleanup step B2. Pooled barcoded libraries (biological triplicates) were sequenced in single lanes using the Illumina NextSeq 500/550 High Output Kit v2 in 75 bp single-end regime.

### RNA-seq

Prior to total RNA extraction, an mRNA spike-in mix composed of four different eukaryotic mRNAs (Plat, Moc, Elav2, and nLuc) was added [46]. These mRNAs were generated by *in vitro* transcription from pJET plasmids using the MEGAscript T7 Transcription Kit (Thermo Fisher Scientific). The amount of RNA spike-in was 10 ng per 30 ml of culture at OD_600_ of 0.5. Each frozen cell pellet was resuspended in 240 μl of TE buffer (pH 8.0) plus 60 μl of LETS buffer (50 mM Tris–HCl pH 8.0, 500 mM LiCl, 50 mM EDTA pH 8.0, and 5% SDS), and 600 μl acidic (pH∼3) phenol/chloroform (1:1). The samples were sonicated in a fume hood, centrifuged and the aqueous phase was extracted two more times with acidic phenol/chloroform and precipitated with ethanol. The RNA was dissolved in double-distilled water and treated with DNase (TURBO DNA-free Kit, Invitrogen). 1 μg of DNase-treated RNA was ribodepleted with a riboPOOL Kit Pan-Actinobacteria (siTOOLs). Sample integrity was checked using an Agilent 2100 Bioanalyzer Pico Chip. The ribodepleted RNA sample (20–100 ng) was used for library construction according to the NEXTFLEX Rapid Directional RNA-Seq Kit. The libraries were checked using an Agilent 2100 Bioanalyzer Nano Chip. The pooled barcoded libraries were sequenced in a single lane with Illumina NextSeq 500/550 High Output Kit v2 in 75 bp single-end regime at the Institute of Molecular Genetics AS CR, Prague, Czech Republic.

### RT-qPCR

5 μl of RNA (∼ 2.5 μg) was reverse transcribed into cDNA (20 μl reaction, SuperScriptIII, Invitrogen) using random hexamers and amplified by RT-qPCR in a LightCycler 480 System (Roche Applied Science) in duplicate reactions containing LightCycler 480 SYBR Green I Master and 0.5 μM primers (each). Gene-specific primers were designed with Primer3, the sequences are listed in the Supplementary Data.

Negative controls (no-template reactions and reactions with RNA as the template to check for genomic DNA contamination) were included in each experiment. The quality of the PCR products was determined by dissociation curve analysis, and primer efficiency was determined by standard curves. Relative mRNA levels were quantified based on threshold cycles (*C*t) for each PCR, normalized to the Plat mRNA spike-in value using the formula 2^(*C*t^(spike)^ − *C*t^(mRNA)^). Relative expression (*E*) was then normalized to the control strain (*E* = * E*_depleted_ /*E*_control_).

### Glycerol gradient ultracentrifugation

*M. smegmatis* exponential and stationary phase cells were pelleted, resuspended in 20 mM Tris–HCl, pH 8, 150 mM KCl, 1 mM MgCl_2_, 1 mM DTT, 0.5 mM PMSF and a Calbiochem Protease Inhibitor Cocktail Set III protease inhibitors, and then sonicated 15 × 10 s with 1 min pauses on ice before being centrifuged. Protein extracts (1 mg) were loaded on a linear 10–30% glycerol gradient, which was prepared in gradient buffer (20 mM Tris–HCl, pH 8, 150 mM KCl, and 1 mM MgCl_2_), and fractionated by centrifugation at 130 000 × *g* for 17 h using an SW-41 rotor (Beckman). The gradient was divided into 20 fractions, and the proteins in each fraction were resolved on SDS–PAGE gels and detected by western blotting.

### ITC measurements

Isothermal titration calorimetry (ITC) experiments were carried out using a MicroCal Auto-iTC200 calorimeter (Malvern Panalytical) at 27°C with stirring at 750 rpm. Both CarD and CrsL were both transferred into identical buffers (50 mM Tris–HCl, 100 mM NaCl, and 0.5 mM DTT, pH 7.5) for the titration purposes. The cell contained 27.6 μM CarD, which was titrated by 315 μM CrsL. Measurements were performed in triplicate, including blank reactions to account for the heat of dilution (buffer:CrsL and CarD: buffer). The titration consisted of 30 injections per measurement, with an initial delay of 120 s. The injection volume was set at 1.3 μl, with 240 s spacing between each step. The raw data were deposited in the Molecular Biophysics Database (MBDB) under entry number 92738-h4q29 and in Zenodo.

The analysis was performed by Microcal PEAQ-ITC Analysis software. The blank measurements were subtracted from the raw data by MEAN subtraction method. The binding isotherms were fitted to the one-site binding model.

### Preparation of isotope-labeled proteins for NMR measurements

[^15^N]-CrsL, [^13^C,^15^N]-CrsL, and [^13^C,^15^N]-CarD were expressed in 2 L of minimal media (M9) containing 1 M MgSO_4_, 500 mM CaCl_2_, 100 mM MnCl_2,_ 50 mM ZnSO_4_, and 50 mM FeCl_3_ [49] and supplemented with ^15^NH_4_Cl and [^13^C_6_]- or unlabeled glucose. The cultures were incubated at 37°C and shaken at 120 rpm for ∼ 3 h (until OD_600_ reached ∼0.6); expression was induced with 0.4 mM IPTG. The temperature was then lowered to room temperature (∼20°C) and the cultures were shaken for an additional 3 h. The proteins were then purified as described above, with an additional size-exclusion chromatography step on a 16/60 Superdex 30 pg (CrsL) or Superdex 75 pg (CarD) column in GF buffer (50mM Tris–HCl, 300 mM NaCl, 1 mM DTT, and 1 mM NaN_3_, pH 7.5). The samples were dialyzed against the NMR buffer: 20 mM sodium phosphate buffer, pH 7, 0.5 mM TCEP and 1 mM NaN_3,_ containing either 10 mM NaCl (low salt) or 300 mM NaCl (high salt). The purified CrsL and CarD were then concentrated using a Vivaspin^®^ 15R Centrifugal Concentrator (Sartorius) with 2000 MWCO and 10 000 MWCO, respectively.

### NMR measurements

All experiments were performed using Bruker Avance III HD 950 MHz and 850 MHz NMR spectrometers equipped with a TCI cryogenic probe head with *z*-axis gradients. The temperature was set to 27°C for the measurements and calibrated according to the chemical shift differences of the pure methanol peaks. All samples contained 10% deuterium dioxide (D_2_O). ^1^H–^15^N correlation was observed in 2D ^1^H–^15^N heteronuclear single-quantum coherence (HSQC) spectra [50, 51]. For backbone resonance assignment, the following samples and the standard set of 3D triple-resonance experiments were used: 0.2 mM [^13^C,^15^N]-CrsL in low-salt NMR buffer, 0.4 mM [^13^C,^15^N]-CrsL with 0.8 mM unlabeled CarD in high-salt NMR buffer, 0.5 mM [^13^C,^15^N]-CarD in high-salt NMR buffer, 0.5 mM [^13^C,^15^N]-CarD with 1 mM unlabeled CrsL in high-salt NMR buffer samples consisting of HNCA [52], HN(CO)CA [53], HNCACB [54], and CBCA(CO)NH [55]. In addition, a ^15^N-edited 3D NOESY spectrum [56] was recorded for 0.4 mM [^13^C,^15^N]-CrsL with 0.8 mM unlabeled CarD. The carbonyl ^13^C chemical shifts for secondary structure propensity (SSP) assessment were obtained from an HNCO spectrum [52] of free [^13^C,^15^N]-CrsL. A 2D ^1^H–^15^N TROSY spectrum [57] was acquired with 256 scans for 1 mM free [^13^C,^15^N]-CarD. Titration experiments were conducted in a high-salt NMR buffer by mixing samples of 0.2 mM [^15^N]-CrsL and 0.2 mM [^15^N]-CrsL with 0.4 mM unlabeled CarD to fully saturate CrsL. 2D [^1^H,^15^N] HSQC spectra of 2 mM [^15^N]-CrsL with 0, 25, 50, 75, 100, 125, 150, 175, 200, 300, and 400 μM CarD were recorded in the titration series. These spectra and their acquisition parameters were deposited in the BioMagResBank (BMRB) under the following entry numbers: 52735 (free CrsL), 53355 (labeled CrsL–CarD complex), 53356 (free CarD), and 53357 (CrsL–CarD labeled complex).

The spectra were processed using the NMRPipe software [58] and the subsequent assignment of the peaks was done using Sparky 3.115 software (T. D. Goddard and D. G. Kneller, University of California). Values of SSP were calculated using the SSP (1.0) script and the chemical shifts of the assigned backbone residues [59]. As a point of reference, we used the predicted chemical shifts of the random coil form of our protein calculated from the sequence using the Poulsen IDP/IUP random coil chemical shifts script [60–62]. Neighbor-corrected structural propensity (ncSP) values were computed using the ncSP calculator [63] with chemical shifts based on those of Tamiola, Acar, and Mulder [64]. Values corresponding to the AlphaFold2-predicted structure were calculated using the SHIFTX2 web tool [65].

### *In silico* predictions

Multiple sequence alignments were constructed based on hits from PHI-BLAST queries and results from DeepMSA (2.0) [66, 67]. We curated putative homologs based on similarity and compared them with the KEGG Sequence Similarity DataBase [68]. The final multiple sequence alignment was prepared using ClustalW in the UGENE toolkit and plotted in ESPript (3.0) [69, 70]. The sequence logo was created using WebLogo (3.7.12) software [71]. Disorder prediction was performed using PSIPRED 4.0, NetSurfP 3.0, IUPred2A, ESpritz 1.3, and flDPnn predictors [72–76]. For IUPred2A, we chose the IUPred2 short disorder option. For predictions using ESpritz, we selected the NMR prediction type and Best Sw as the decision threshold option. The structural prediction of CrsL from *M. smegmatis* and its heterodimer predictions with CarD or RNAP were computed using AlphaFold2 and AlphaFold Multimer extensions [77, 78].

### NGS data processing and analysis

#### ChIP-seq

The ChIP-seq peak-calling and gene-assignment procedures were performed as previously described [44]. Briefly, the reads were mapped to the *M. smegmatis* genome (NCBI RefSeq NC_008596.1) using HISAT2 [79], and peaks were called by MACS2 [80]. Venn diagrams showing the overlap of peaks were created using the BEDTools intersect [81] and matplotlib-venn package (https://pypi.org/project/matplotlib-venn/). The three-way Venn diagram was created with Intervene [82]. Coverage profiles of the 1 kb region around the ORF start were generated using the deepTools [83] (computeMatrix, plotProfile), with programming libraries rtracklayer [84], Pandas (https://pandas.pydata.org/), Matplotlib [85], and Seaborn [86].

#### RNA-seq

The read quality was checked using version 0.11.9 of FastQC (https://www.bioinformatics.babraham.ac.uk/projects/fastqc/). Where necessary, adapters and low-quality sequences were removed using Trimmomatic 0.39 [87]. The reads were then aligned to the reference genome using HISAT2 2.2.1 [88] and SAMtools 1.13 [89, 90]. Read coverage tracks were computed using deepTools 3.5.1 [83]. The DESeq2 R package [91] was used to identify differentially expressed genes (DEGs) at FDR ≤ 0.05.

## Results

### CrsL binds to CarD in *M. smegmatis*

**Figure 1:**
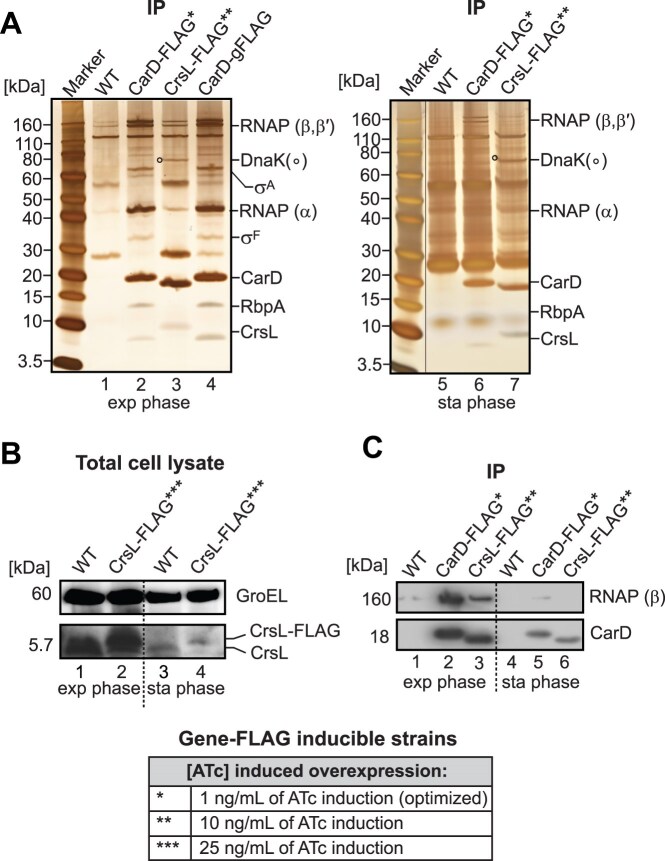
(**A**) Co-immunoprecipitated proteins from CarD–FLAG* (1 ng/ml ATc) and CrsL-FLAG** (10 ng/ml ATc) strains, collected at both exponential and stationary phases, as well as from the CarD–gFLAG strain (exponential phase only), were resolved on SDS–PAGE and visualized by silver staining. The identity of the protein bands was confirmed by MALDI-FTICR mass spectrometry. Note that the presence of a FLAG tag slows down the migration of the tagged protein compared to the nontagged protein. (**B**) Western blot of total cell lysates from wt (WT) and CrsL-FLAG*** (25 ng/ml ATc) in the exponential and stationary phases. CrsL levels were detected by anti-CrsL antibody. GroEL was used as loading control and detected with an anti-GroEL antibody. (**C**) The immunoprecipitated proteins from the CarD–FLAG* and CrsL–FLAG** strains were detected by western blotting with anti-RNAP (β) and anti-CarD antibodies. Asterisks indicate the different ATc concentrations (ng/ml) used in the inducible FLAG-tagged strains.

Our search for CarD interacting partners began with an *M. smegmatis* strain containing an additional copy of FLAG-tagged CarD under an ATc-inducible promoter [92]. Immunoprecipitation of CarD–FLAG from cells in the exponential and stationary phases revealed its association with RNAP subunits, σ^A^, RbpA and, notably, a protein encoded by the *MSMEG_5890* gene (Fig. 1A, lane 2). *MSMEG_5890* encodes a small protein (predicted MW 5.7 kDa), which we named “CrsL” (CarD RNA polymerase small linker). The function of CrsL is unknown.

To confirm the CarD–CrsL interaction, we generated a strain in which the FLAG-tag sequence was inserted into the *M. smegmatis* genome at the *carD* locus (CarD–gFLAG). CarD–gFLAG immunoprecipitation confirmed the interaction of CarD with CrsL and other known CarD interaction partners: σ^B^ and WhiA [11, 93–96] (Fig. 1A, lane 4, and Supplementary Fig. S1A, lane 3). We also observed an association between CarD–gFLAG and ApeB (*MSMEG_5828*), a putative protease homologous of *M. tuberculosis* PepC [97, 98], in stationary phase (Supplementary Fig. S1A, lane 3). However, while CarD level decreased in the wild-type (wt) strain during stationary phase, CarD–gFLAG levels remained high, although CarD–gFLAG is expressed from its endogenous promoter (Supplementary Fig. S1B, lanes 11 and 12). The antisense RNA of *carD* (*AscarD* RNA) was recently shown to be expressed in stationary phase and to negatively regulate CarD expression [38]. In the CarD–gFLAG constructed strain, adding the sequence that encodes the FLAG-tag and the hygromycin resistance cassette to the *carD* genome locus (Supplementary Fig. S1C) disrupted the expression of *AscarD* RNA. This resulted in an increased level of the CarD protein. Although we confirmed that ApeB interacts with elevated levels of CarD in the stationary phase (Supplementary Fig. S1D), we were not able to reciprocally confirm the CarD–ApeB interaction using the endogenous FLAG-tagged ApeB under normal conditions (Supplementary Fig. S1A, lane 2, and Supplementary Fig. S1E). Therefore, ApeB binds to CarD only when its level is increased, and the level of CarD can affect its interacting proteins.

Unlike ApeB, CrsL interacted with CarD not only when the CarD levels were elevated (Fig. 1A and Supplementary Fig. S1A) but also when CarD–FLAG levels were optimized to be comparable to the endogenous CarD levels in the wt strain (Fig. 1A, lanes 2 and 6, and Supplementary Fig. S1B, lanes 15 and 16). Under these conditions, CrsL peptides were highly abundant in CarD–FLAG immunoprecipitates in both the exponential and stationary phases, as detected by mass spectrometry (Supplementary Table S1).

To confirm the interaction between CrsL and CarD, we constructed a strain with FLAG-tagged CrsL under an ATc inducible promoter [92]. We generated our own mouse anti-CrsL antibody and verified the CrsL–FLAG expression. Even at an induction level of 25 ng/ml ATc (indicated by ***), CrsL–FLAG levels were comparable to those of the endogenous CrsL protein in the wt strain (Fig. 1B, lane 1 versus 2, Supplementary Fig. S1F). CrsL–FLAG** immunoprecipitated CarD from cells in both exponential and stationary phases, as detected by mass spectrometry (Fig. 1A, lanes 3 and 7), and by western blotting with the anti-CarD antibody (Fig. 1C). Additionally, RNAP β, β’ and α subunits co-immunoprecipitated with CrsL–FLAG** in exponential but not stationary phase (Fig. 1A, lanes 3 and 7 and Fig. 1C, lanes 3 and 6). In both growth phases, CrsL interacted with the DnaK chaperon protein (*MSMEG_0709*) (Fig. 1A, lanes 3 and 7).

In conclusion, we have identified a novel small protein that we have named CrsL. CrsL binds to the essential mycobacterial transcription factor CarD in both the exponential and stationary phases.

### CrsL is evolutionarily conserved in actinobacteria

**Figure 2.**
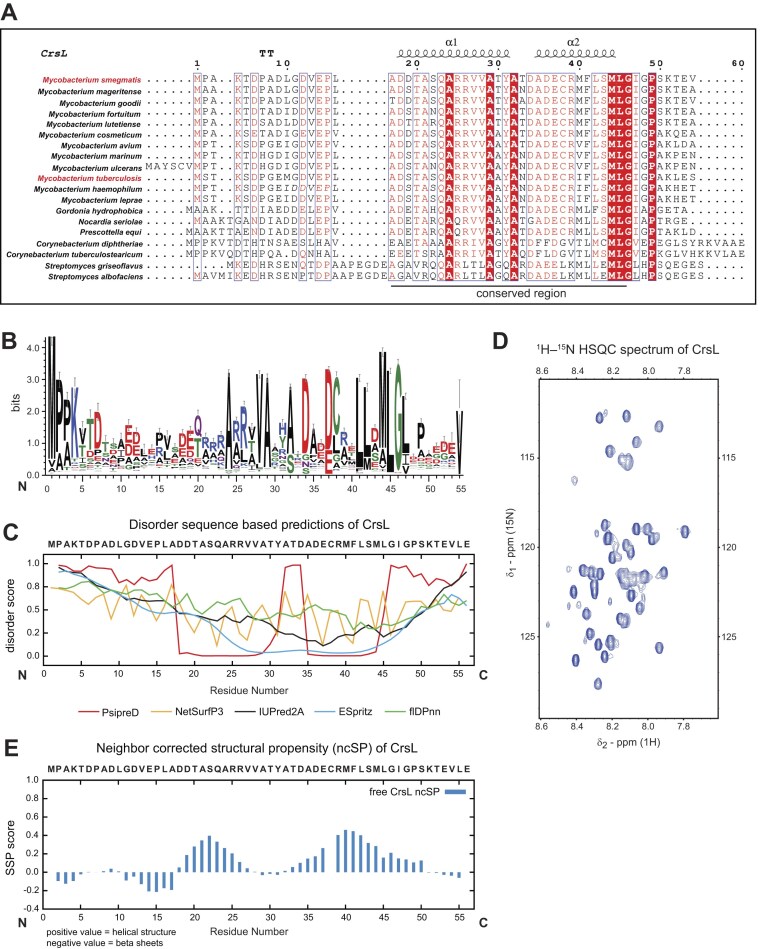
(**A**) Multiple sequence alignment of selected CrsL homologs in actinobacteria. Conserved regions and the secondary structure elements are depicted above the alignment. The protein names are listed in Supplementary Table S2. (**B**) Multiple Sequence Alignment of the CrsL sequence by DeepMSA2 showing the highly conserved region (20–45 residues) within 770 entries of homologous bacterial sequences. (**C**) Disorder sequence-based prediction of CrsL using different web tools. Tools’ names are indicated below the figure. Values above 0.4 are considered as intrinsically disordered regions. The CrsL protein does not have an ordered structure with exceptions of the PsipreD and ESpritz predictions. (**D**) The 2D ^1^H–^15^N HSQC spectrum of CrsL. The proton resonances have a narrow distribution pointing toward a disordered state of the protein. (**E**) ncSP of CrsL calculated from the chemical shifts of the protein backbone. Relatively low values point to a disordered state with a slight propensity toward two α-helical regions. Cysteine residues (as Cys38 in CrsL) are not computed by the ncSP default settings.

To further characterize CrsL, we explored the evolutionary relationship between its homologs across diverse bacterial species. We conducted a multiple sequence alignment of selected CrsL homologs, identifying the 20–45 aa residue region as the most conserved part of CrsL (Fig. 2A and B, and Supplementary Table S2). CrsL is conserved in many actinobacterial species, including *Mycobacteria, Nocardia, Streptomyces, Corynebacteria*, and *Rhodococcus* (Fig. 2A and Supplementary Table S3). CrsL homologs are found in species such as *M. tuberculosis* (*Rv3489*), *M. bovis* (*Mb3519*), and *M. marinum* (*MMAR_4977*), where they are annotated as hypothetical proteins with an unknown function [99, 100].

### CrsL is an intrinsically disordered protein

To probe the secondary structures of CrsL and obtain insights into its potential three-dimensional arrangement, we first used protein structure predictions. Combined results from several prediction tools, namely PsipreD [72], NetSurfP3 [73], IUPred2A [74], ESpritz [75], and flDPnn [76], showed low values for secondary structures. Three of these tools (NetSurfP3, flDPnn, and IUPred2A) consistently assigned ambiguous values to most residues. On the contrary, PsipreD and ESpritz predicted two conserved regions of CrsL (aa 20–27 and 34–48) to be well-ordered (Fig. 2C). Similarly, AlphaFold2 [77] predicted these two regions to be structured (Supplementary Fig. S2A). This suggests the formation of ordered structures in complexes with interacting partner(s).

Next, we purified CrsL labeled with ^13^C and ^15^N isotopes, measured its NMR spectra, and performed backbone assignment. We successfully identified all of the peaks corresponding to the atoms forming the CrsL backbone. The 2D ^1^H–^15^N HSQC spectra were used to monitor the individual amide-proton signals of the protein (Fig. 2D). Based on the spectrum obtained, the proton resonances exhibit a narrow distribution typical of many intrinsically disordered proteins, including residues forming the two regions predicted to be structured (Fig. 2C). Furthermore, we computed the ncSP and SSPs to obtain more detailed structural information. The low ncSP/SSP values (Fig. 2E and Supplementary Fig. S2B) indicate that free CrsL is an intrinsically disordered protein (IDP) with a propensity of 20–40% to form α-helical structures in the Asp19–Arg25 and Glu37–Met44 regions. IDPs are mostly characterized in eukaryotes, where they often serve as scaffolds for multiprotein interactions. Nevertheless, intrinsically disordered protein domains are also involved in interactions affecting bacterial transcription [101, 102].

### CrsL binds directly and tightly to CarD

**Figure 3.**
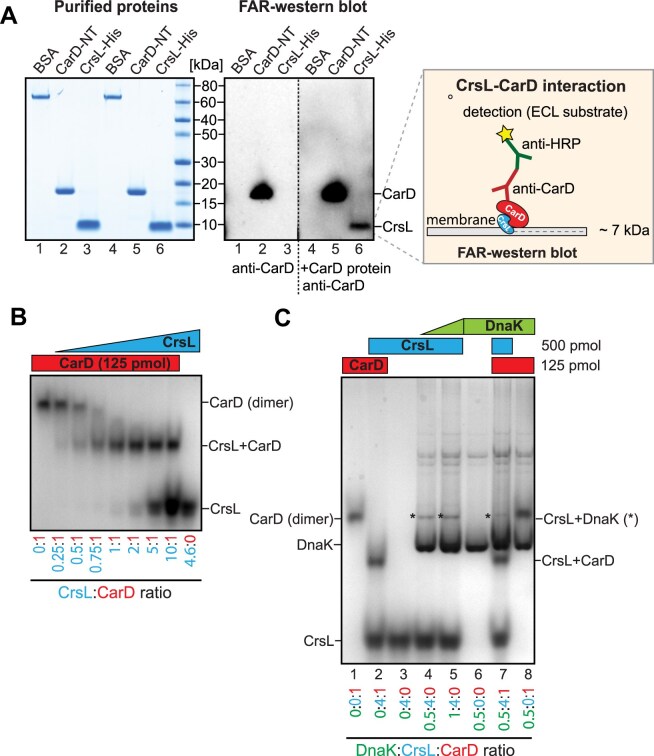
(**A**) The direct interaction between CrsL and CarD was detected *in vitro* using FAR western blotting with purified CarD–NT and CrsL–His proteins and an anti-CarD antibody. The purified proteins were resolved on SDS–PAGE and stained with Coomassie. BSA and a membrane incubated without the CarD protein were used as negative controls. (**B**) Native gels with CarD and CrsL *in vitro* purified proteins. CarD and CrsL were mixed in the ratios indicated below the gel and separated on 8% PAGE. The gels were stained with Coomassie, and the identity of the protein bands was confirmed by mass spectrometry. In (**C**), DnaK protein was additionally added to the mixture in the ratios indicated below the gel followed by a similar experiment as described in B.

To determine whether the CrsL–CarD interaction is direct or mediated by another protein, we performed FAR-western blotting [48] using *in vitro* purified CarD and CrsL proteins. With the anti-CarD antibody, we detected CarD associated with the nitrocellulose membrane-bound CrsL (Fig. 3A, lane 6), indicating that CrsL binds directly to CarD.

To further characterize the CarD–CrsL complex, we performed native gel electrophoresis to monitor the formation of the complex using *in vitro* purified CrsL and CarD proteins (Fig. 3B). CarD alone migrated as a dimer; however, upon the addition of CrsL, the CarD dimer dissociated and CarD bound to CrsL. We then determined the dissociation constant (*K*_d_​) of the CarD–CrsL complex using isothermal titration calorimetry (ITC, Supplementary Fig. S2C). In three independent measurements, the *K*_d_​​ values were in the nanomolar range (4.3, 4.5, and 3.3 nM, respectively). A *K*_d_​​​ of ~4 nM indicates a strong interaction. This interaction is enthalpy-driven (Δ*H* = −21 kcal/mol), associated with a large entropic cost (−*T*Δ*S* = 9 kcal/mol). The results indicate a binding stoichiometry of one CrsL molecule to one CarD subunit.

While nearly all CrsL and CarD were associated, only a minor fraction of DnaK interacted with 62.5 nM CrsL *in vitro* (Fig. 3C), suggesting that the CrsL–DnaK interaction is considerably weaker. This is consistent with the observation that DnaK, which also binds CrsL *in vivo*, did not inhibit the CrsL–CarD association (Fig. 3C, lane 7). We confirmed that CrsL directly binds to both CarD and DnaK. However, the CrsL–CarD interaction is significantly stronger.

### CrsL is folded upon CarD binding

**Figure 4.**
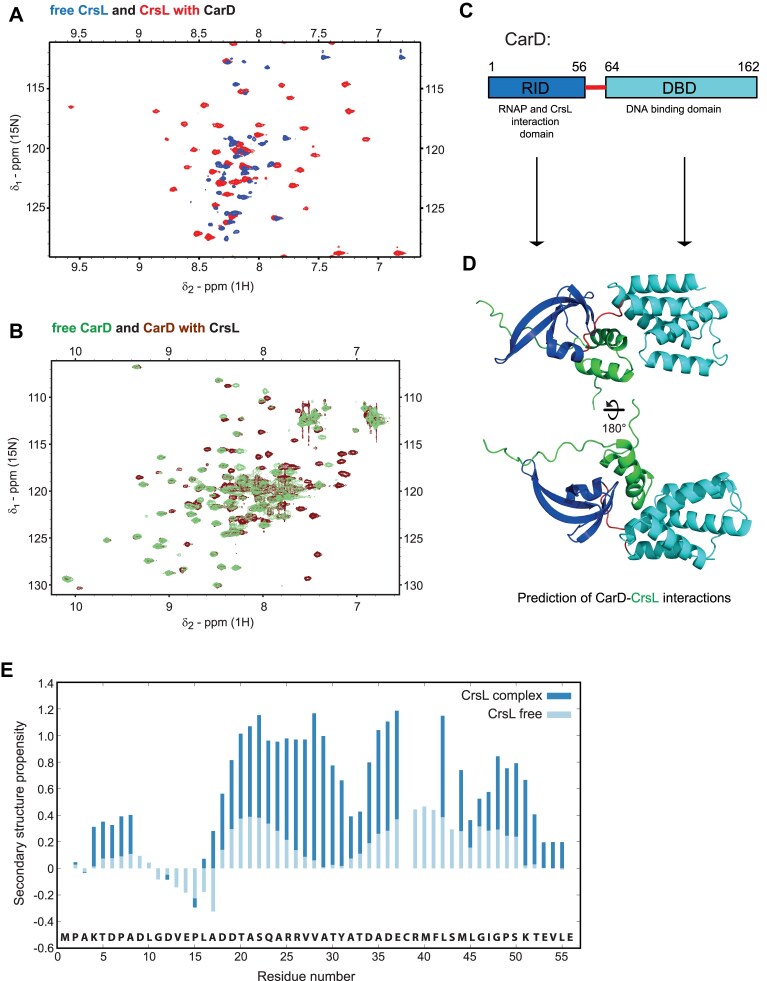
(**A**) Spectral overlay between 2D ^1^H–^15^N HSQC spectrum of free CrsL (blue) and the spectrum of CrsL bound to CarD (red). (**B**) Spectral overlay of the 2D ^1^H–^15^N HSQC spectrum of free CarD (mint green) and complex with CrsL (maroon). (**C**) Scheme of CarD architecture, with the N-terminal domain responsible for CrsL and RNAP binding (RID), and C-terminal DNA interaction domain (DBD). (**D**) An AlphaFold Multimer model of the interaction between CarD N-terminal domain (blue), loop (red), CTD (cyan), and CrsL (green). The prediction positions CrsL between the two domains, consistently with the NMR experiments. (**E**) Neighbor corrected Structural Propensity was calculated exclusively from C-α chemical shifts for the complex (dark blue) due to signal broadening in the central region. A shift toward more pronounced positive values suggests that CrsL adopts a helix-turn-helix motif upon binding to CarD. The stabilization of the secondary structure is apparent, in contrast to the relatively low values of the unbound CrsL (light blue).

To gain structural insight into the interaction, we recorded 2D ^1^H–^15^N HSQC spectra of [^15^N]-CrsL with increasing concentration of CarD (selected peaks in detail in Supplementary Fig. S3). The peaks of [^15^N]-CrsL bound to CarD exhibited large chemical shift dispersion, which is typical of well-ordered proteins (Fig. 4A). The positions of free or bound [^15^N]-CrsL peaks did not change during titration, as expected for a nanomolar *K*_d_, indicating slow binding. However, the peaks of the complex with chemical shifts influenced by the CarD binding were very broad, indicating conformational and/or chemical exchange influences between the bound and free form. The exchange broadening was significantly suppressed by increasing the NaCl concentration to 300 mM, revealing stabilization of the complex by enhancing hydrophobic interactions (Supplementary Fig. S4A).

The suppressed broadening allowed us to assign the majority of peaks using triple resonance and ^15^N-edited NOESY spectra recorded on [^13^C,^15^N]-CrsL with an excess of unlabeled CarD. The peaks of Thr5–Ala17 and Thr52–Val54 were sharp, showing that the terminal regions remained disordered in the complex. However, the peaks of most residues were broad in the ^1^H–^15^N HSQC spectra and invisible in HNCACB and CBCA(CO)NH spectra, making the assignment difficult especially for the Thr33–Leu45 residues. The spectral features described suggest that CrsL in complex with CarD is mostly folded, yet exhibiting considerable exchange dynamics.

The changes observed in the [^13^C,^15^N]-CarD spectra upon CrsL binding (Fig. 4B and Supplementary Fig. S4B) are consistent with those described above. Whereas peaks from the N-terminal RNA interaction domain (RID) were only observed (and assigned using triple-resonance NMR experiments) in 2D ^1^H–^15^N HSQC or TROSY spectra of free [^13^C,^15^N]-CarD, additional peaks appeared upon the addition of unlabeled CrsL, with intensities comparable to peaks of [^15^N]-CrsL residues in the complex with unlabeled CarD. The reduced peak broadening of [^13^C,^15^N]-CarD in the presence of CrsL clearly shows that binding to CrsL substantially alters the structure of CarD, which is also consistent with the results of native gel electrophoresis indicating dissociation of the CarD dimer upon CrsL binding. The chemical shifts assigned to free and bound CarD are identical for residues Ile3–Thr46 in the RID and Gly58–Arg60 in the linker between the RID and the CTD (Fig. 4C), but differ for residues Asp61–Gly67 in the linker region. This suggests that the RID adopts a rigid structure that is identical in the free dimeric and CrsL-bound forms of CarD, and that the linker region is involved in CarD binding. The number of peaks observable only in complex with CrsL indicates that they belong mostly to residues of CTD, but the intensity of the corresponding peaks in triple-resonance experiments is insufficient for a reliable assignment.

The structure of the CrsL–CarD complex predicted by AlphaFold Multimer [77, 78] is in a good agreement with the NMR data (Fig. 4D and Supplementary Fig. S5). The CrsL residues Ala17–Leu45 are folded in a helix-turn-helix motif, with the turn formed by Ala32–Asp34. The SSP, calculated from ^13^C chemical shifts available for most CrsL residues, shows the formation of α-helices between Thr20 and Ile47, interrupted in the same region as predicted by AlphaFold Multimer (Fig. 4E and Supplementary Fig. S2B). In the predicted complex structure, CrsL binds to the pocket between C-terminal (CTD) and N-terminal (RID) domains of CarD (Fig. 4D and Supplementary Fig. S5). The predicted binding site includes the highly conserved loop V56–V62 (Supplementary Fig. S6) and overlaps with the interaction surface of CarD in its complex with RNAP [103]. This suggests that CrsL interferes with the CarD–RNAP interaction. On the other hand, the predicted binding site is far from the C-terminal motif of CarD recognized by the Clp protease [104].

In conclusion, NMR data and AlphaFold Multimer predictions provide evidence that CrsL forms a well-ordered helix-turn-helix motif upon binding to CarD, causes dissociation of the CarD dimer, and most likely interferes with the CarD–RNAP interaction.

### The majority of CrsL co-sediments with CarD

In the exponential phase, CrsL and RNAP were co-immunoprecipitated with CarD–FLAG (Fig. 1A). Reciprocally, CarD and RNAP were co-immunoprecipitated with CrsL–FLAG (Fig. 1C). To determine whether CrsL primarily associates with CarD or RNAP, we performed glycerol gradient ultracentrifugation using exponential and stationary phase *M. smegmatis* lysates. The gradient was then fractionated and the distributions of CrsL, RNAP, σ^A^, CarD, and RbpA were analyzed by western blotting.

In exponential phase, CrsL mainly sedimented in the top fractions (1-5) where CarD was also predominantly found (Supplementary Fig. S7A). Fractions 4 and 5 contained RNAP, but the majority of CrsL co-sedimented with CarD in fractions 1–3. Only a subset of CrsL molecules co-sedimented with both CarD and RNAP in fractions 4 and 5, indicating that the majority of CrsL is not bound to RNAP.

In stationary phase, CrsL was not detected in the top fractions where most of the CarD sedimented. CrsL sedimented in the bottom fraction together with a minor amount of CarD (fraction 20) (Supplementary Fig. S7A). Although *M. tuberculosis* RNAP can oligomerize *in vitro* to form supramolecular complexes [105] and RNAP was partially detected in the bottom fraction of the gradient (Supplementary Fig. S7A), RNAP was not co-immunoprecipitated with CrsL–FLAG in the stationary phase (Fig. 1A, lane 7). This suggests that CrsL likely participates in other complexes that do not include RNAP, possibly involving the DnaK chaperone, a major CrsL-interacting protein (along with CarD), in the stationary phase (Fig. 1A, lane 7).

### CrsL level is decreased in stationary phase and is dependent on CarD

CrsL predominantly binds to CarD and changes conformation upon CarD binding. We, therefore, asked whether CarD might affect CrsL stability *in vivo* and if CrsL levels correlate with CarD expression.

Both CarD and CrsL protein levels decreased in the stationary phase (Fig. 1B and Supplementary Figs S1B and 7B). CrsL levels increased when CarD was artificially elevated in the CarD–gFLAG strain during stationary phase (Supplementary Fig. S7B, lane 8). To further confirm that the level of the CarD protein alters the level of the CrsL protein, we generated strains containing an ATc-inducible CRISPR system [106] to knockdown CarD, which is an essential protein [33]. Upon the addition of ATc, the mRNA and protein levels of the *carD* gene were efficiently depleted (∼90% depletion) in both the exponential and stationary phases (Supplementary Fig. S7C and D). CrsL protein levels were reduced upon CarD depletion (Supplementary Fig. S7D and E), indicating that the amount of CrsL depends on CarD.

Consistent with this, CrsL was more abundant in CarD–FLAG co-immunoprecipitations during the exponential phase, when the level of CarD increases, than during the stationary phase (Fig. 1A, lane 2 versus lane 6). Similarly, elevating CarD levels in the CarD–gFLAG strain during the stationary phase increased the amount of CrsL detected in CarD–FLAG co-immunoprecipitations (Supplementary Fig. S7F, lane 6 versus lane 8), consistent with a positive correlation between CarD and CrsL protein levels.

In contrast, the knockdown of the *crsL* gene did not affect the level of CarD (Supplementary Figs S7D and 6E). However, we note that although *csrL* mRNA was efficiently depleted (>90%), at the protein level, CrsL was depleted by 70% and 50% in the exponential and stationary phases (Supplementary Fig. S7E). Taken together, the CarD level affects the CrsL protein level, and the formation of the CarD–CrsL complex is mainly regulated by the levels of the interacting proteins during cell growth.

### CrsL is a potential mycobacterial transcription factor

As CrsL was found in a complex with RNAP and CarD during the exponential phase (Fig. 1A), we used chromatin immunoprecipitation sequencing (ChIP-seq) with CrsL–FLAG to determine if CrsL is associated with the bacterial chromosome. Additionally, we compared the genomic binding sites of CrsL with those of CarD, RbpA, RNAP, and σ^A^/σ^B^ [44, 46] under the same experimental conditions. All ChIP-seq data and detected peaks are available on the msmegseq.elixir-czech.cz webpage, which has an integrated IGV genome browser [107].

**Figure 5.**
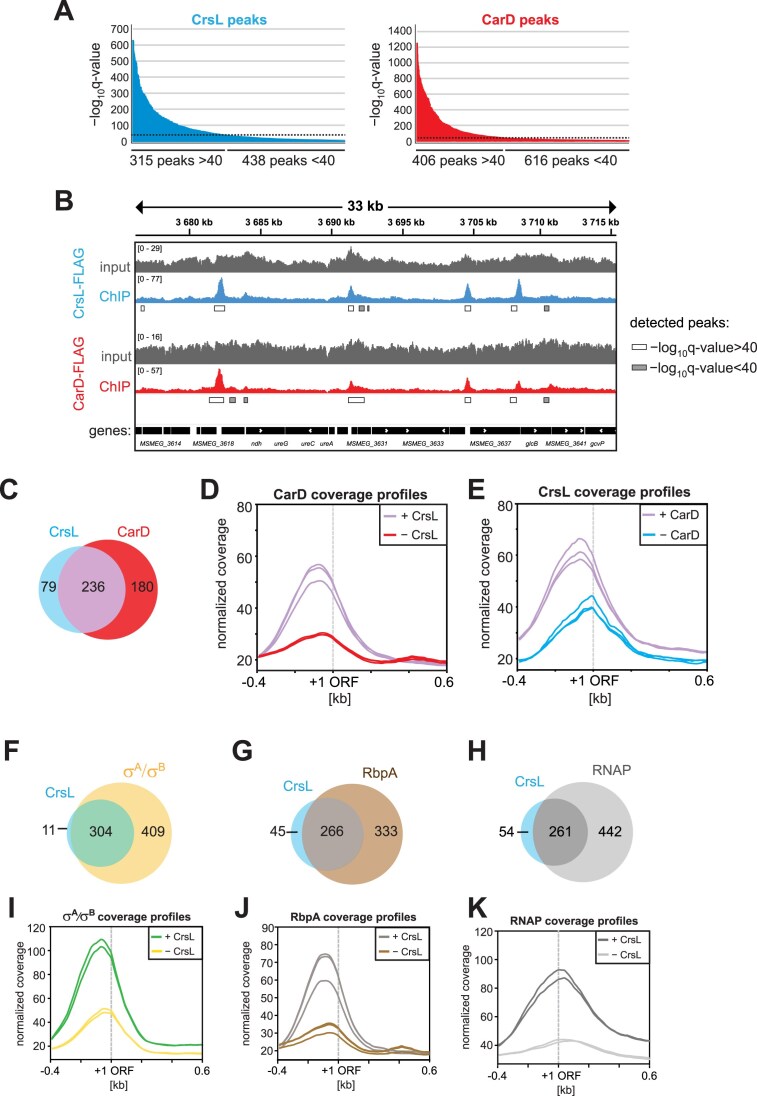
**A**) ChIP-seq peaks detected with CrsL–FLAG and CarD–FLAG. The experiments were done in biological triplicates. The peaks are divided according to a significancy cutoff set as 40 and the higher −log_10_  *q*-value value indicates higher statistical significancy of the peaks. (**B**) An example of ChIP-seq detected peaks for CrsL–FLAG versus CarD–FLAG within a 33 kb region. The significance of the peaks is indicated by boxes (white/gray) below the detected peaks. (**C**) A Venn diagram showing the overlap of CrsL- and CarD-binding sites derived from the ChIP-seq peaks. (**D**) ChIP-seq coverage profiles of CarD for genes either associated with both CarD and CrsL (+CrsL) or genes associated only with CarD (−CrsL) in a 1 kb region around ORF for individual samples (lines). Genes significantly associated with both CarD and CrsL at their promoters tend to have higher CarD peak coverage than those lacking CrsL association (purple lines). (**E**) ChIP-seq coverage profiles of CrsL for genes either associated with both CrsL and CarD (+CarD) or genes associated only with CrsL (−CarD) in a 1 kb region around the ORF for individual samples (lines). Genes significantly associated with both CrsL and CarD at their promoters tend to have higher CarD peak coverage than those lacking CrsL association (purple lines). (**F**) Venn diagram showing the overlap of CrsL and σ^A^/σ^B^ -binding sites derived from ChIP-seq peaks. (**G**) Venn diagram showing the overlap of CrsL- and RbpA-binding sites derived from ChIP-seq peaks. (**H**) Venn diagram showing the overlap of CrsL and RNAP binding sites derived from ChIP-seq peaks. (**I**) ChIP-seq coverage profiles of σ^A^/σ^B^ for genes either associated with both σ^A^/σ^B^ and CrsL (+CrsL) or genes associated only with σ^A^/σ^B^ (−CrsL) in a 1 kb region around the ORF for individual samples (lines). (**J**) ChIP-seq coverage profiles of RbpA for genes either associated with both RbpA and CrsL (+CrsL) or genes associated only with RbpA (−CrsL) in a 1 kb region around the ORF for individual samples (lines). (**K**) ChIP-seq coverage profiles of RNAP for genes either associated with both RNAP and CrsL (+CrsL) or genes associated only with RNAP (−CrsL) in a 1 kb region around the ORF for individual samples (lines).

The results showed that CrsL is indeed associated with the genome. We detected comparable numbers of CrsL peaks in the *M. smegmatis* genome relative to CarD peaks (315 versus 406, Fig. 5A and Supplementary Table S4). CrsL and CarD peaks were highly enriched at the 5′ ends of genes, regions that correspond to gene promoters. An example of representative ChIP-seq data is shown in Fig. 5B.

Around 75% of the significant peaks (−log_10_  *q*-value > 40) detected for CrsL overlapped with CarD peaks (Fig. 5C). Especially promoter regions with high CarD occupancy were almost always associated with CrsL (Fig. 5D). Likewise, promoter regions with high CrsL occupancy were associated with CarD (Fig. 5E). This positive correlation between CrsL and CarD suggests that promoters interacting with CarD are more likely to also be associated with CrsL, and vice versa. Nevertheless, both CrsL and CarD can also interact with promoters in the absence of each other (Fig. 5C, blue versus red colors).

In the ChIP-seq data, only 79 of CrsL-associated genomic loci were not bound by CarD (Fig. 5C). However, we noticed that 56 of the 79 CrsL peaks overlap with low-significant CarD peaks (−log_10_  *q*-value < 40) that were originally excluded from the analysis. The remaining subset of 23 CrsL peaks does not overlap with any detectable CarD peaks but exclusively overlaps with σ^A^/σ^B^ peaks (no RNAP, CarD, and RbpA peaks). Two examples are the promoters of the genes *MSMEG_1821* (encoding acyl-CoA dehydrogenase) or *MSMEG_5136* (annotated as helix-turn-helix motif protein) (Supplementary Fig. S8A).

The majority of CrsL-bound genomic loci were also associated with σ^A^/σ^B^, RbpA, and RNAP (Fig. 5F–H, respectively). Moreover, promoters associated with CrsL displayed higher coverage of σ^A^/σ^B^, RbpA, and RNAP peaks than promoters without CrsL (Fig. 5I–K, respectively). Interestingly, across the entire *M. smegmatis* genome, we identified only 11 CrsL peaks that did not overlap with σ^A^/σ^B^ (Fig. 5F). This suggests that CrsL predominantly associates with the promoter regions of σ^A^/σ^B^-dependent genes (Fig. 5I).

**Figure 6.**
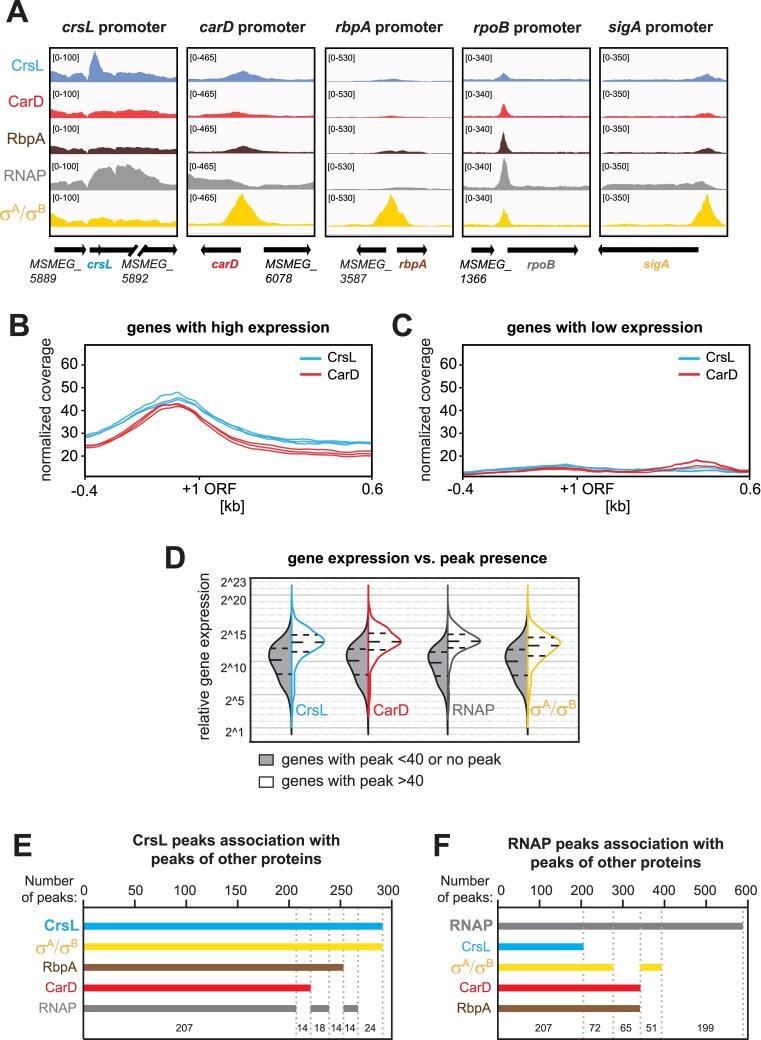
**A**) ChIP-seq detected peaks for CrsL, CarD, RbpA, RNAP and σ^A^/σ^B^ on their promoters. For comparison, the peak heights for all datasets were set to the same value based on the highest peak score. The average of CrsL (in blue) and CarD (in red) peak profiles (each line represents one biological replicate) over 200 genes with the highest expression (**B**) and 200 genes with no or lowest expression (**C**) in the exponential phase of *M. smegmatis* [41] . The 1000 bp region is shown with +1 representing the start site of the ORF and −0.4 kb upstream and +0.6 kb downstream of the ORF. (**D**) Violin plots showing the relative gene expression levels of genes with/without significant CrsL, CarD, RNAP, and σ^A^/σ^B^ association. The white color (right sides) of violin plots shows the expression of genes with peaks −log_10_  *q*-value > 40 while the gray color (left sides) of the violin plots shows expression of genes with no or less significant peaks (−log_10_  *q*-value < 40). Dashed lines show the lower and upper quartiles. (**E**) Plot showing intersections of CrsL peaks with peaks of σ^A^/σ^B^ or RbpA or CarD or RNAP. CrsL predominantly associates with the majority of gene promoters with a peak profile intersects with CarD, RbpA, RNAP, and more with σ^A^/σ^B^. (**F**) Plot showing intersections of RNAP peaks with peaks of CrsL or σ^A^/σ^B^ or CarD or RbpA. The 199 RNAP peaks that are not present in CrsL, RbpA, CarD, and σ^A^/σ^B^ likely represent those peaks of RNAP located within gene regions or at the 3′ ends of genes. The major set of RNAP peaks (*n* = 207) overlaps with peaks of CarD, RbpA, σ^A^/σ^B^, and CrsL.

The CrsL peaks, similar to CarD peaks, were detected at the promoters of genes involved in transcription regulation, including the *carD, rpoB*, and *sigA* genes (Fig. 6A). CrsL also binds to its own promoter (Fig. 6A) and rRNA promoters (Supplementary Fig. S8B). It also binds to tRNA genes and ribosomal protein-coding genes (Supplementary Table S4). Based on Gene Ontology Term analysis [108], many CrsL-bound genes are involved in protein biosynthesis, amino-acid biosynthesis, and tricarboxylic acid cycle biological processes. We generated average ChIP-seq profiles of CrsL and CarD for 200 highly expressed genes as well as for 200 genes with low to no expression in the exponential phase [41] (Fig. 6B and C, respectively; for the expression profiles of the two gene groups see Supplementary Fig. S8C). CrsL was associated with highly expressed genes and was notably absent from nontranscribed or lowly expressed genes, as was CarD (Fig. 6B and C). Globally, CrsL interacted with the promoters of actively transcribed genes similarly to CarD, RNAP, or σ^A^/σ^B^ (Fig. 6D) and the majority of CrsL-associated promoters interacted also with CarD, RNAP, RbpA, and σ^A^/σ^B^ (Fig. 6E and F).

We have identified the binding motif for CrsL in the DNA which resembles a promoter (Supplementary Fig. S9A and B). These data suggest that CrsL is a transcription factor that generally binds to the promoters of highly expressed genes in *M. smegmatis*.

### Identification of CrsL- and CarD-regulated genes in *M. smegmatis*

To determine the effects of *crsL* depletion on the *M. smegmatis* transcriptome, we performed RNA-seq using the *crsL* knockdown strain in both the exponential and stationary phases. In parallel, we performed RNA-seq with the *carD* knockdown strain to compare the DEGs regulated by both CrsL and CarD.

**Figure 7.**
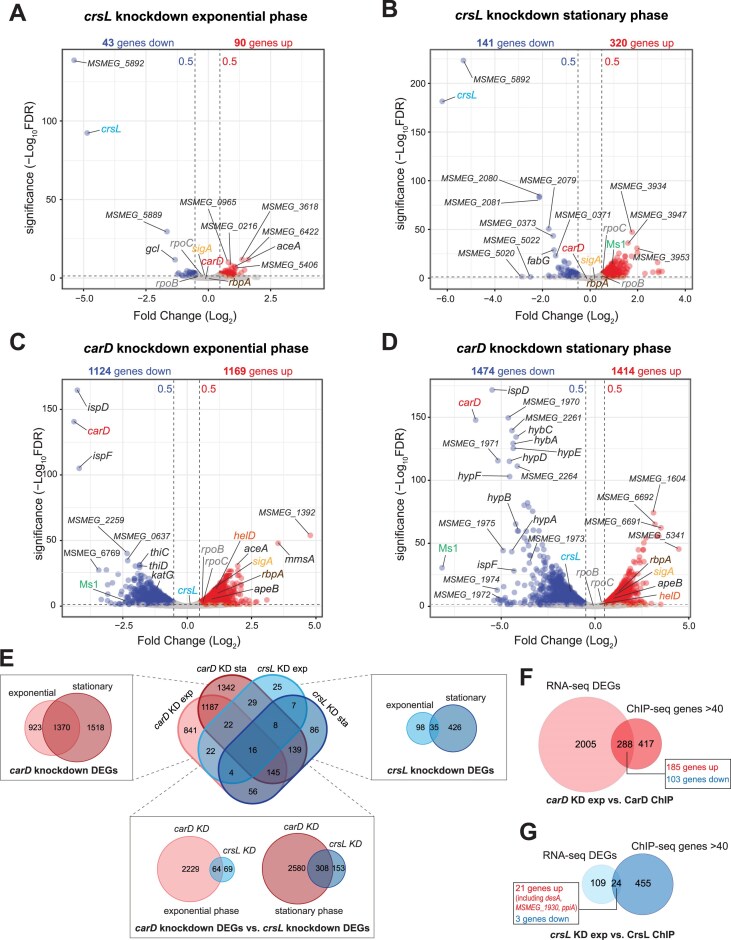
Volcano plots showing the fold change (FC, log_2_) against significance (−log_10_FDR corrected *P*-value) of transcripts identified by RNA-seq analysis of *crsL* knockdown or *carD* knockdown in exponential phase (**A** and **C**, respectively) and *crsL* knockdown or *carD* knockdown in stationary phase (**B** and **D**, respectively). Blue dots indicate significant genes with an FC ≤ −0.5; red dots indicate significant genes with an FC ≥ 0.5; gray dots indicate unchanged genes. The significancy cutoff (−log_10_FDR corrected *P*-value) was set to 1.3 (i.e. FDR ≤ 0.05). (**E**) Venn diagrams showing the overlap of DEGs between the *carD* and *crsL* knockdown datasets. (**F**) Venn diagrams showing overlap of *crsL* knockdown exponential phase DEGs with the CrsL ChIP-seq associated genes. (**G**) Venn diagrams showing the overlap of *carD* knockdown exponential phase DEGs with the CarD ChIP-seq associated genes.

After CrsL depletion in exponential phase, relatively few genes were affected (|Log_2_FC| ≥ 0.5, FDR-corrected *p*-value ≤ 0.05). 90 genes significantly increased expression, while 43 genes decreased expression (Fig. 7A). In the stationary phase, 320 genes significantly increased expression while 141 genes decreased expression (Fig. 7B). In either phase, CrsL depletion did not affect the *carD* mRNA level. The relatively small number of affected genes could be due to the less efficient depletion of the CrsL protein. The DEGs likely represent the tip of the iceberg, the genes requiring CrsL the most for their regulation.

On the contrary, nearly one-third of the genes in the genome changed expression after CarD depletion: 1169 genes significantly increased expression while 1124 genes decreased expression in the exponential phase (Fig. 7C and Supplementary Table S5). In stationary phase, 1414 genes significantly increased expression while 1474 genes, including *crsL*, decreased expression (Fig. 7D). Interestingly, the most affected gene in the stationary phase was Ms1, a binding partner of the RNAP core [40, 41]. The effects of CarD and CrsL depletions in the stationary phase were unexpected, as their protein levels are already highly reduced compared to the exponential phase (Fig. 1B and Supplementary Fig. S1B).

When we compared the genes regulated by CrsL and CarD, 64 genes were affected by both CrsL and CarD depletion in the exponential phase (∼50% of the *crsL* DEGs) (Fig. 7E). In the stationary phase, 308 genes were affected by both CrsL and CarD depletion (∼66% of the *crsL* DEGs) (Fig. 7E).

Most of the DEGs are indirectly regulated by CrsL and CarD in the exponential phase. ChIP-seq detected CrsL association with 24 out of the 133 genes that changed expression upon CrsL depletion (Fig. 7F and Supplementary Table S6). A similar scenario was observed for CarD - 288 genes (out of 2293 genes that changed expression upon CarD depletion) were associated with CarD (Fig. 7G and Supplementary Table S6). Additionally, many promoters were occupied by CrsL or CarD, but the expression of the genes under these promoters was not altered by CrsL or CarD depletion, respectively (Fig. 7F and G).

CrsL affects gene expression in both the exponential and stationary phases, with a greater number of genes altered during the stationary phase. For the majority of genes directly regulated by CrsL (21 out of 24), CrsL acts as a repressor.

### CrsL alters CarD association with RNA polymerase *in vivo*

**Figure 8.**
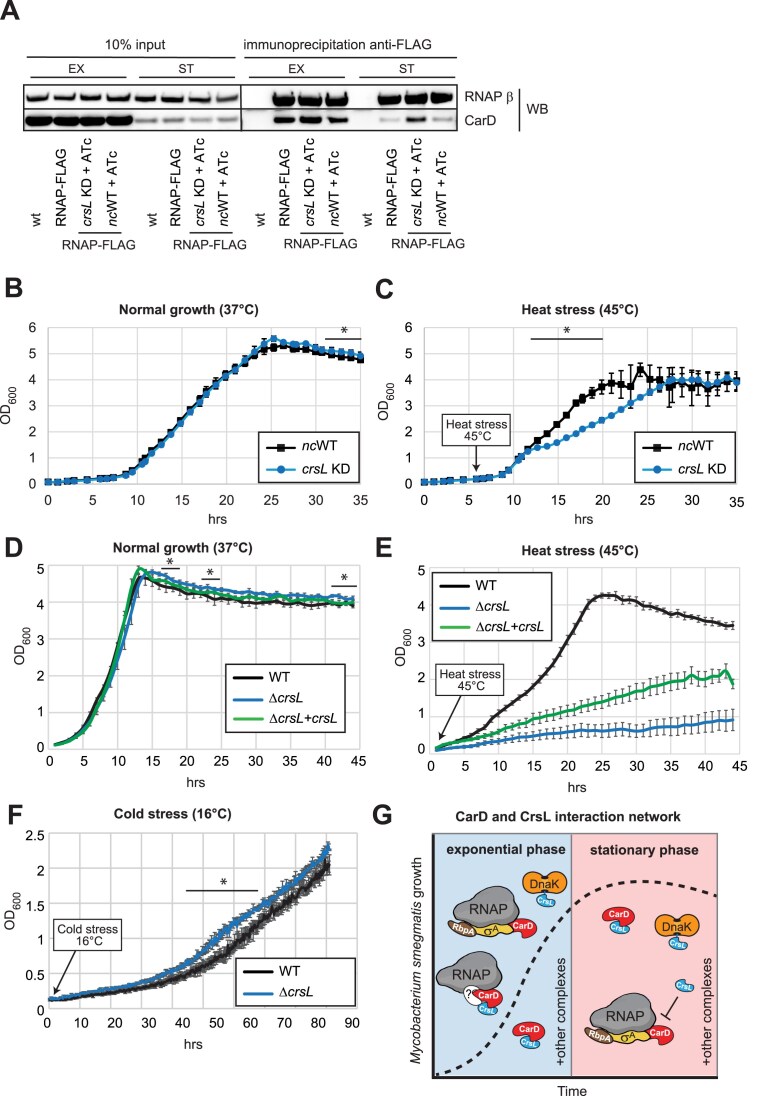
**A**) A RNAP-FLAG-tagged *crsL* depletion strain (*crsL* KD) was used to assess the effect of CrsL on the amount of CarD bound to RNAP in exponential and stationary phases. Endogenously FLAG-tagged RNAP was pulled down via FLAG, and the co-immunoprecipitated CarD was detected by western blotting using anti-RNAP (β) and anti-CarD antibodies. “wt” indicates the negative control strain with untagged RNAP, all other strains carried FLAG-tagged RNAP (labeled “RNAP-FLAG”). “*crsL* KD” is the *crsL* depletion strain; “*nc*WT” is the strain with a negative control gRNA (with no homologous sequence in the *M. smegmatis* genome). gRNA (guide RNA) expression (CrsL depletion) was induced with 100 ng/ml anhydrotetracycline (“+ATc”). (**B** and **C**) Growth curves of the *crsL* depletion strain (*crsL* KD) versus the negative control (*nc*WT) strain from biological triplicates incubated at 37°C (B) or at 45°C (C). (**D** and **E**) Growth curves of the ∆*crsL* strain, the ∆*crsL* + *crsL* strain and the wt strain from biological triplicates incubated at 37°C (D) or at 45°C (E). (**F**) Growth curves of the ∆*crsL* strain and wt strain from biological triplicates incubated at 16°C. Asterisks in (B), (C), (D), and (F) indicate a statistically significant change between the wt and the *csrL*-depleted or ∆*crsL* strain (paired *t*-test, *P* < 0.05). The cells were grown in a Biosan RTS-8 Multi-Channel Bioreactor and OD_600_ was measured over the growth. Error bars represent SEM. (**G**) Schematic representation of the CarD and CrsL interaction network from our study.

RNA-seq analysis of the *crsL*-depleted strain indicates that CrsL functions more frequently as a repressor. At the same time, CrsL binds to the promoters of highly expressed, CarD-associated genes. CrsL interacts strongly with CarD, and only a small fraction of CrsL is associated with RNAP. AlphaFold Multimer predictions further suggest that CrsL influences CarD binding to RNAP (Supplementary Fig. S9C and D), consistent with our NMR measurements showing that CrsL binds near the CarD domain interacting with RNAP (Fig. 4). Together, these findings suggest a role for CrsL in regulating CarD–RNAP association. To test this, we utilized a *crsL* knockdown strain introduced into a background expressing endogenously FLAG-tagged β′ subunit of RNAP and measured the amount of CarD bound to RNAP after the CrsL depletion. We did not detect any considerable changes during the exponential phase; however, the amount of CarD bound to RNAP increased after *crsL* knockdown in the stationary phase (Fig. 8A). Therefore, CrsL negatively affects the CarD–RNAP interaction in the stationary phase, which is consistent with the markedly higher number of DEGs following CrsL depletion during this growth phase (Fig. 7B). As CarD levels decrease in the stationary phase, CrsL may have a greater impact on CarD association with RNAP and thereby negatively regulate gene expression.

To further assess the role of CrsL in stationary phase, we compared the growth curves of CrsL-depleted cells (*crsL* knockdown) to those of the control strain (*nc*WT) at 37°C. The *crsL* knockdown strain showed a modest but reproducible increase in optical density during the stationary phase compared to the control strain (Fig. 8B). These results suggest that CrsL contributes to the bacterial response to nutrient-limiting conditions during the stationary phase.

### CrsL affects *M. smegmatis* growth at elevated temperature

Our data showed that CrsL directly regulates the expression of the *MSMEG_5773* (encoding DesA, fatty acid desaturase) and *MSMEG_1930* genes (encoding DEAD/DEAH box RNA helicase). The DesA enzyme introduces double bonds into fatty acid chains, producing unsaturated fatty acids. This process is crucial for enhancing membrane fluidity at low temperature [109–114]. The DEAD/DEAH RNA box helicase remodels the RNA secondary structures formed during cold stress [115–119]. CrsL acts as a repressor for these genes. It associates with their promoters, and the expression of both genes increased following CrsL depletion. Therefore, CrsL may influence growth at both elevated and reduced temperatures.

To test the role of CrsL under elevated temperatures, we measured the growth of CrsL-depleted cells (*crsL* knockdown) compared to the control (*nc*WT) strain at 45°C (heat stress was induced during the mid-exponential phase). CrsL-depleted cells showed slower growth compared to the control (Fig. 8C).

The CrsL-encoding gene is organized in the two-gene *MSMEG_5890-MSMEG_5892* operon and was proposed to be transcribed from a single promoter located upstream of *crsL* [120]. This organization is highly conserved in other mycobacterial species, including *M. tuberculosis* [121] (Supplementary Fig. S10A). The second gene of the operon, *MSMEG_5892* (*otsA*), is not essential in *M. smegmatis* [122, 123] and encodes α,α-trehalose-phosphate synthase. Trehalose, a disaccharide important for the biosynthesis of the mycobacterial cell wall [124–126], also functions as an osmoprotectant during heat stress. RNA-seq analysis following *crsL* knockdown showed decreased expression of both genes in this operon (Fig. 7A and B).

Therefore, to confirm the results from the *crsL* knockdown strain, we also generated a ∆*crsL* strain, in which the *crsL* gene was replaced with a hygromycin resistance gene. Additionally, we constructed a complementation strain of the deleted gene (∆*crsL* + *crsL* strain), in which an extra copy of the *crsL* gene under its native promoter was integrated into the genome at the L5 mycobacteriophage *attB* site. We then measured the growth of these strains compared to the wt control strain at 37°C and 45°C (Fig. 8D and E). The ∆*crsL* strain showed reduced growth at the elevated temperature, similar to the *crsL* depletion strain (Fig. 8C), confirming CrsL’s role in heat stress adaptation. The ∆*crsL* + *crsL* strain was partially able to rescue growth at 45°C, although the growth rate was still not comparable to that of the wt. Interestingly, the ∆*crsL* + *crsL* strain behaved similarly to the wt during the first 5 h of heat stress.

The complemented *crsL* gene is no longer part of the native operon with *otsA*. We confirmed that the *crsL* gene is expressed in the ∆*crsL* + *crsL* strain, but the levels of *crsL* mRNA differ between the wt and the ∆*crsL* + *crsL* strain (Supplementary Fig. S10B). RNA-seq data indicated that *crsL* may also be transcribed as part of a second operon together with the upstream gene *MSMEG_5889*, from an alternative promoter that we named P0 (Supplementary Fig. S10C). We confirmed that *crsL* is also transcribed from the P0 promoter (Supplementary Fig. S10D). In the ∆*crsL* + *crsL* strain, the *crsL* gene was cloned together with its upstream promoter P1, but the distant P0 promoter was not included, which might affect the growth of the ∆*crsL* + *crsL* strain at 45°C.

Then, we used the ∆*crsL* and wt strains to test whether CrsL affects the growth at cold temperatures. In contrast to heat stress, the ∆*crsL* strain exhibited enhanced growth at 16°C compared to the wt control (Fig. 8F). This phenotype is consistent with RNA-seq data showing that CrsL represses *desA* and *MSMEG_1930*, genes important for growth at low temperatures. Thus, while CrsL promotes survival under heat stress, it limits growth under cold stress, highlighting its key role in fine-tuning bacterial adaptation across diverse temperature conditions.

We conclude that CrsL is a novel transcription regulator that interacts with CarD and RNAP, influencing the CarD–RNAP complex formation (Fig. 8G), and is conserved among actinobacterial species. CrsL regulates gene expression during both growth phases. Its regulon largely overlaps with that of CarD. Importantly, CrsL specifically controls genes required for survival under temperature stress, enabling *M. smegmatis* to adapt to both low and high temperatures.

## Discussion

In this study, we expanded the interaction network of the essential mycobacterial transcription factor, CarD, by identifying two new interacting partners, CrsL and ApeB, in *M. smegmatis*.

ApeB is a putative aminopeptidase that cleaves proteins from the amino terminus. The specific function of ApeB and its homolog in *M. tuberculosis* is currently unknown. However, our data suggest that ApeB interacts with CarD only under conditions where CarD levels are elevated.

In contrast to ApeB, CrsL binds to the cellular levels of CarD. The CrsL–CarD interaction is direct, and both proteins associate with RNAP. What is CrsL’s role in mycobacteria? CrsL is conserved among actinobacteria. In *M. tuberculosis*, the *crsL* homolog (*Rv3489*) is not essential [127, 128] and its expression is predicted to be regulated by IdeR [129], an iron-dependent regulator that represses genes involved in iron uptake and maintains iron homeostasis. In *M. bovis* BCG, the expression of the *crsL* homolog (*BCG_3553*) is activated by the RaaS (regulator of antimicrobial-assisted survival) transcription factor, which is important for mycobacterial long-term survival [130]. Therefore, CrsL may play a role in adapting to environmental changes, such as fluctuating iron levels or conditions associated with long-term growth.

In *M. smegmatis*, CRISPR-mediated depletion of *crsL* mRNA was highly effective (almost 99%), yet the reduction in CrsL protein was suboptimal (Supplementary Fig. S7E), indicating that CrsL is regulated post-transcriptionally. Bacteria appear to compensate for the decreased level of *crsL* mRNA by enhancing its translation or stabilizing the CrsL protein. The level of CrsL protein depends on CarD, which is also regulated post-transcriptionally by an antisense RNA of *carD* (*AscarD* RNA) and the Clp protease [38]. These findings suggest that the bacteria actively monitor CarD and CrsL levels to maintain their optimal levels necessary for gene expression.

ChIP-seq data revealed that CrsL binding sites in the *M. smegmatis* genome overlap with those of CarD as well as RbpA, σ^A^/σ^B^, and RNAP (Fig. 5). CrsL predominantly binds to promoter regions, and the motif enriched in CrsL-bound regions resembles promoter sequences. CrsL showed a similar binding pattern to CarD, and both proteins preferentially interacted with actively transcribed genes. Considering the interaction of CrsL with CarD and their similar expression profiles during *M. smegmatis* growth, this suggests a cooperative role in gene expression regulation. Partial depletion of CrsL altered the expression of numerous genes, although this effect may be underestimated due to residual CrsL protein remaining in the cells. Notably, genes affected by CrsL depletion overlap in part with those influenced by CarD depletion. Collectively, the data indicate that CrsL is a component of the mycobacterial transcriptional machinery.

Although CrsL, CarD, RbpA, σ^A^/σ^B^, and RNAP proteins can bind to the same promoters, it is possible that their binding is sequential rather than simultaneous, and they are not present at the same promoter at the same time. CarD is recruited to the promoter with RNAP, where it stabilizes the open promoter complex during transcription initiation. CrsL appears to negatively affect the CarD–RNAP interaction. ChIP-seq data may capture transient CrsL–RNAP–CarD intermediates. Afterward, CarD dissociates from RNAP but remains bound to CrsL. Previous studies have shown that the regulatory effect of CarD depends on the promoter sequence, with CarD activating transcription at unstable promoters but repressing it at promoters with stable open complexes [32, 35]. Thus, CrsL could fine-tune transcriptional responses by modulating the CarD–RNAP interaction through this kinetic mechanism.

In RNA-seq data, the *otsA* (*MSMEG_5892)* expression also decreased following CrsL depletion (Fig. 7A and B), potentially due to dCas9 recruitment to the *crsL* gene locus within the *crsL*–*otsA* operon [41, 106]. *otsA* encodes an enzyme in the conserved trehalose biosynthetic pathway, OtsA/B [131] and has not been reported to regulate transcription. This suggests that the DEGs identified by RNA-seq are primarily regulated by CrsL. Trehalose protects biological molecules against abiotic stresses. OtsA has been associated with enhanced viability upon desiccation in *Rhizobium etli* [132] and with the survival of *Salmonella enterica* at 50°C [133]. While most prokaryotes possess only the OtsA/B pathway, mycobacteria have the OtsA/B, TreY/TreZ, and TreS enzymes for trehalose synthesis [125]. In *M. smegmatis*, these three pathways are functionally redundant, with trehalose levels in the Δ*otsA* mutant are comparable to those in the wild-type [124]. Furthermore, the Δ*otsA* shows no growth impairment at 43°C. These findings strongly indicate that the reduced growth observed upon CrsL depletion at elevated temperatures is primarily due to CrsL, not OtsA. In addition, the Δ*crsL* strain showed also reduced growth at 45°C confirming the role of CrsL in heat stress adaptation (Fig. 8E). Nevertheless, the organization of the *crsL–otsA* operon is conserved among actinobacteria, suggesting that it has been maintained for its functional or regulatory advantages.

We focused specifically on the direct targets of CrsL (Fig. 7G) and identified two genes linked to temperature-sensitive phenotype: DesA (*MSMEG_5773*) is a desaturase enzyme involved in fatty acid biosynthesis, which contributes to membrane fluidity in cold stress [109, 134, 135]. The DEAD/DEAH box helicase (*MSMEG_1930*) is an RNA chaperone that remodels RNA structures and RNA–protein complexes in an ATP-dependent manner, supporting essential processes in RNA metabolism, including transcription, RNA processing, translation, and RNA decay [116, 136–138]. Under cold stress, double-stranded RNA secondary structures are formed in bacteria [139, 140] and the expression of *MSMEG_1930* is highly upregulated in the early stages of cold stress in *M. smegmatis* [141]. CrsL directly regulates the expression of *desA* and *MSMEG_1930* by binding to their promoters. When CrsL is depleted, expression of these genes increases. This regulatory effect is reflected in the growth phenotype: the Δ*crsL* strain grows better at 16°C than the wt (Fig. 8F), most likely because higher expression of *desA* and *MSMEG_1930* provides an advantage under cold conditions. Conversely, under heat stress (45°C), both the CrsL-depleted strain and the Δ*crsL* strain display impaired growth relative to the wt (Fig. 8C and E), suggesting that uncontrolled expression of *desA* and *MSMEG_1930* becomes detrimental at high temperatures. Together, these data demonstrate that CrsL, which regulates genes involved in adaptation to temperature changes, is essential for bacterial growth under temperature-stress conditions.

Besides CarD and RNAP, CrsL also interacts with the DnaK protein (Fig. 1A). DnaK (Hsp70) is an essential chaperone in *M. smegmatis* [142]. Mycobacterial DnaK is involved in the native folding of important proteins, such as RNAP subunits. It also interacts with mutated, rifampicin-resistant RNAP β subunits, which increases antibiotic resistance [143]. Future studies will determine whether CrsL could affect the DnaK–RNAP interaction.

As part of the Hsp70 family of heat shock proteins, *dnaK* expression increases at elevated temperatures [144]. DnaK prevents protein aggregation, assists in the refolding of denatured proteins, and maintains protein quality control [145–148]. In *E. coli*, DnaK regulates the availability of σ^32^, which controls the expression of heat shock response genes. Under normal conditions, DnaK associates with σ^32^, preventing σ^32^ binding to the promoters. During heat shock, accumulated denatured proteins sequester DnaK, releasing σ^32^, which then activates the expression of heat shock response genes, including *dnaK* itself. As DnaK levels increase and the amount of denatured proteins decreases, DnaK again sequesters σ^32^, shutting down σ^32^-dependent transcription [149]. However, the molecular mechanisms regulating heat shock genes differ considerably among bacterial species. In mycobacteria, DnaK is synthesized from the *dnaKJE-hspR* operon, which is autoregulated by the HspR repressor [144]. HspR has a C-terminal hydrophobic tail that is the primary site where DnaK binds [150]. DnaK enhances the DNA-binding activity of HspR [151]. Here, we demonstrate that DnaK interacts with CrsL, which helps bacteria adapt to temperature shifts and modulates the binding of the general mycobacterial transcription factor CarD to RNAP. Thus, CrsL represents a novel link between the DnaK heat shock protein and the regulation of the transcriptional machinery.

CrsL contains a stretch of “MLGIGP” amino acids in its sequence, which is highly conserved and can be considered as hydrophobic. In addition, CrsL appears to be an intrinsically disordered protein on its own. It is plausible that DnaK interacting with CrsL affects its folding or function. The structure of CrsL, or its interaction with DnaK (whose availability is regulated by temperature shifts and the levels of denatured proteins) could act as an additional sensor for temperature fluctuations.

Additional genes regulated by CrsL are also linked to temperature changes and protein folding. Based on RNA-seq data, CrsL is a repressor for the *MSMEG_0024* (Rv0009 in *M. tuberculosis*) encoding PpiA, peptidyl-prolyl *cis–trans* isomerase B (Fig. 7G). These enzymes specifically catalyze the *cis–trans* isomerization of peptide bonds at proline residues, accelerating protein folding and enhancing folding efficiency. Interestingly, in *M. tuberculosis*, PpiA is repressed by HrcA [144], the second transcriptional repressor controlling heat shock genes (including *groEL2* and *groES* chaperones).

Interestingly, the *MSMEG_0373* promoter is exclusively associated with CrsL and σ^A^, but not with RNAP, CarD, and RbpA (Supplementary Fig. S8A). The *MSMEG_0373* homolog in *M. tuberculosis* is fadA2 (*Rv0243*). This membrane-anchored enzyme catalyzes the final step of β-oxidation in the fatty acid degradation pathway. This pathway involves several steps to break down fatty acid molecules into acetyl-CoA, which is then utilized in the tricarboxylic acid cycle [152]. FadA2 is one of six putative thiolases involved in the final step of β-oxidation. Primary σ factors, such as σ^A^, typically cannot stably bind to promoters on their own. Although we only observed CrsL and σ^A^ at the *MSMEG_0373* promoter by ChIP-seq, we cannot exclude the presence of additional proteins that might associate with CrsL and σ^A^ in a larger complex. Mycobacterial σ^A^ interacts with several transcription factors, including PhoP or CRP (cAMP receptor protein, Crp1, *MSMEG_6189*) [47]. CRP is a transcriptional regulator that controls gene expression by recognizing altered cAMP levels in bacteria. We propose that there may be alternative, noncanonical mechanisms for regulating σ^A^-dependent transcription in mycobacteria, which are rare, but still present and should be considered.

Our results show that CrsL is intrinsically disordered and unable to form tertiary structures under normal conditions, but it adopts a well-ordered structure upon binding CarD. Many such proteins have been identified over the past three decades [153, 154]. Despite lacking a defined secondary structure, these proteins remain functional and interact with various partners, serving as hub proteins that facilitate molecular communication through protein-protein interactions [155, 156]. Moreover, many disordered proteins exist in a transient state with preformed structural motifs [157, 158]. These proteins can also adopt a defined fold upon binding with other protein(s), both in prokaryotes [157, 159] and in eukaryotes [160–162]. In CrsL, two regions (aa 20–27 and 34–48) are predicted to be potentially structured. A CrsL–CarD interaction predicted by AlphaFold Multimer and characterized experimentally by NMR showed that these regions are really folded in the complex (Fig. 4 and Supplementary Fig. S2B). A comparison with the structure of the promoter melting intermediate with CarD bound to RNAP [103] suggests that CrsL may compete with RNAP for the CarD-binding site. This was confirmed by an *in vivo* experiment showing that CrsL decreases CarD association with RNAP in the stationary phase of growth (Fig. 8A). The unstructured nature of CrsL might enable its rapid association with target protein(s) (swift binding) and allow flexible interaction with diverse binding partners, making CrsL well-suited for sensing and responding to temperature-based shifts. In conclusion, this work identifies CrsL as a mycobacterial transcription factor conserved among actinobacteria. It also provides a foundation for future studies focusing on the molecular details of CrsL’s interaction with CarD/RNAP, its mechanistic role, and its impact on cell physiology, including the regulation of genes involved in temperature adaptation.

## Supplementary Material

gkaf1342_Supplemental_Files

## Data Availability

The sequencing data are available at ArrayExpress: *M. smegmatis* RNA-seq for *carD* and *crsL* knockdown [E-MTAB-13812] and *M. smegmatis* CrsL ChIP-seq [E-MTAB-12348]. All original code has been deposited on Zenodo under DOI:10.5281/zenodo.11174175. Our webpage (msmegseq.elixir-czech.cz) can be used to visualize all ChIP-seq and RNA-seq datasets. The mass spectrometry proteomics data have been deposited to the ProteomeXchange Consortium via the PRIDE partner repository with the dataset identifier PXD058166. The NMR data have been deposited in the BioMagResBank (BMRB) under the following entry numbers: 52735 (free CrsL), 53355 (labeled CrsL–CarD complex), 53356 (free CarD), and 53357 (CrsL–CarD labeled complex). The raw data were deposited in the Molecular Biophysics Database (MBDB) under entry number 92738-h4q29 and in Zenodo.
