# Supplementary material for: Expanding the CarD interaction network: CrsL is a novel transcription regulator in actinobacteria
Source: Nucleic Acids Res. 2025 Dec 17;53(22):gkaf1342. doi: 10.1093/nar/gkaf1342 (PMC12709188; doi:10.1093/nar/gkaf1342)
Supplement: gkaf1342_Supplemental_Files [file gkaf1342_supplemental_files.zip › Supplementary_data_final.pdf]

Expanding the CarD interaction network: CrsL is a novel transcription regulator in actinobacteria

SUPPLEMENTARY TABLES

Supplementary Table 1. Peptides identified in CarD-FLAG* and ApeB-FLAG** immunoprecipitates samples versus wild type in exponential and stationary phase. The complete mass spectrometry proteomics data have been deposited to the ProteomeXchange Consortium PRIDE, accession number PXD058166.

Supplementary Table 2. The alignment of 19 CrsL putative protein homologous sequences used in Figure 2A and of 19 CarD homologous sequences used in Supplementary Figure 6, respectively. The aligments were produced by DeepMSA2.

Supplementary Table 3. The alignment of 770 CrsL putative protein homologous sequences by DeepMSA2 which were used to create a logo in Figure 2B.

Supplementary Table 4. CrsL ChIP-seq data, including peaks with low probabilities, column "ppval" (peak p-value) shows $-\log_{10}$ q-value. Peaks with $-\log_{10}$ q-value >40 were used for further analysis.

Supplementary Table 5. RNA-seq data from CrsL- and CarD-depleted strains compared to the negative control strain in the exponential and stationary phases of growth. Only statistically significant differentially expressed genes are shown (FDR-corrected p-value < 0.05). Genes encoding rRNAs were omitted because the samples were rRNA depleted before RNA-seq.

Supplementary Table 6. The list of statistically significant differentially expressed genes (FDR-corrected p-value < 0.05) after CarD depletion in the exponential phase of growth, which are associated with CarD peaks based on ChIP-seq data from the exponential phase of growth, is shown (289 genes, genes encoding rRNAs were omitted because the samples were rRNA depleted before RNA-seq). The same list is also shown for CrsL (24 genes).

SUPPLEMENTARY MATERIALS AND METHODS

Construction of the bacterial strains

CrsL-6xHis and DnaK-His strains: The CrsL-6xHis (at its N-terminus) was generated by PCR using Q5 High-Fidelity DNA Polymerase (NEB) and *M. smegmatis* genomic DNA (*LK2980*) as template. The PCR product was cloned into pET22b plasmid via NdeI/XhoI restriction sites resulting in *LK3496* (*E. coli* DH5α), verified by sequencing and transformed into *E. coli* DE3 cells, resulting in *LK3499* strain.

The DnaK-His strain was generated by PCR using Phusion High-Fidelity DNA Polymerase (NEB) and *M. smegmatis* genomic DNA (*LK2980*) as template. The PCR product was cloned into pET22b plasmid via NdeI/HindIII restriction sites resulting in *JH183* (*E. coli* DH5α), verified by sequencing and transformed into *E. coli* DE3 cells, resulting in *JH185* strain. The CarD-NT expression strain (*LK3209*) was constructed previously (1).

ApeB-FLAG and CrsL-FLAG strains: The ApeB (*MSMEG_5828*) and CrsL (*MSMEG_5890*) genes were amplified by PCR using Q5 High-Fidelity DNA Polymerase (NEB) with primers #3415 + #3417 (for ApeB), and #3909 + #3910 (for CrsL) and *M. smegmatis* genomic DNA as template. The C-terminal 1× FLAG-tag (DYKDDDDK) was encoded within the reverse PCR primers. CrsL-FLAG was cloned into pTetInt integrative plasmid (2) via NdeI/HindIII restriction sites and verified by sequencing. The plasmids were integrated into *M. smegmatis* mc^2 155 genome by electroporation resulting in ApeB-FLAG (*LK2767*) and CrsL-FLAG (*LK3051*) strains. The CarD-FLAG (*LK2539*), RbpA-FLAG (*LK2541*), HelD-FLAG (*LK2589*) strains were generated previously (1,3).

ApeB-gFLAG and CarD-gFLAG strains: The strains with the FLAG-tagged in the genome (native locus) − ApeB-gFLAG (*LK2846*) and CarD-gFLAG (*LK2899*) were designed using NEBuilder Assembly Tool (https://nebuilder.neb.com/) and constructed using Gibson assembly kit (NEB) as following. The cassettes were generated by assembling three PCR fragments amplified with Q5 High-Fidelity DNA Polymerase (NEB); i) 500 bp long fragment [left arm, LA] homologous to the 3' terminal part of ApeB (*MSMEG_5828*) or CarD (*MSMEG_6077*) containing the FLAG-tag (DYKDDDDK) amplified by primer #3378 + #3379 or #3372 + #3373, respectively; ii) 500 bp long fragment [right arm, RA] homologous to the 5' end of the ApeB or CarD gene amplified by primer #3382 + #3383 or #3376 + #3377, respectively; iii) The left and right arms of ApeB and CarD were flanked by hygromycin resistance cassette amplified from *LK1463* strain by primer #3380 + #3381 or #3374 + #3375, respectively. The three fragments (LA, HYG, RA) of ApeB or CarD were assembled into pUC18 (*LK222*) plasmid with *HindIII*/*XbaI* or *HindIII*/*BamHI*, respectively, using Gibson assembly Cloning kit (NEB). The Gibson assembly constructs were transformed into *E. coli* DH5α. The resulting ApeB (*LK2796*) and CarD (*LK2795*) strains were verified by sequencing. The fragments encompassing the cassettes were subsequently transformed into the *M. smegmatis* pJV53 (*LK1321*) or *M. smegmatis* pYS1 (*LK2713*) strains for homologous recombination and individual clones were selected, resulting in chromosomal FLAG-tagged gene encoding ApeB-gFLAG (*LK2846*) and CarD-gFLAG (*LK2899*) strains.

carD and crsL CRISPR depletion strains: The sgRNA oligonucleotides are listed in Supplementary Data. The sgRNA targeting either the gene (*crsL*) or the gene promoter (*carD*) were cloned into PLJR962 according to the previously established protocol (4), verified by sequencing and transformed by electroporation into *M. smegmatis* wild type (*LK2598)* resulting in strains *LK3301* (*MSMEG_6077*,

carD sgRNA) and *LK3302* (*MSMEG_5890*, *crsL* sgRNA). The negative control non-targeting control sgRNA strain (*nc*WT, *LK2261*) was generated previously (4,5). The same plasmids with cloned sgRNA targeting the *crsl* gene and the non-targeting control sgRNA were transformed by electroporation into *M. smegmatis* RNAP-FLAG strain (*JH7*, described previously (1)). The resulting strains (*JH151*, *JH171*) were used for immunoprecipitation experiments.

Δ*crsL* (*MSMEG_5890*) *strain:* To prepare the *crsL* deletion strain, the homologous recombination was used to replace the *MSMEG_5890* coding sequence from the start codon to the stop codon with hygromycin resistance gene. The following DNA fragments were prepared by PCR using Phusion DNA polymerase (NEB): the left arm and the right arm for homologous recombination (550 bp upstream of the start codon and 550 bp downstream of the stop codon of the *MSMEG_5890* gene, respectively) and the hygromycin resistance gene (6). The pUC18 plasmid was linearized using SphI and BamHI restriction enzymes. The three PCR fragments and the linearized pUC18 were combined in a Gibson assembly reaction. The assembled DNA was then transformed to *E. coli* DH5α competent cells and the resulting construct (*JH359*) was verified by sequencing. Next, the cassette for recombination containing the left homology arm, the hygromycin resistance gene and the right homology arm was digested from the construct by HindIII and KpnI restriction enzymes. The linear cassette was then electroporated to *M. smegmatis* expressing the pJV53 plasmid with increased homologous recombination frequency (7). Individual clones were selected for hygromycin resistance. Next, cells were cured from the pVJ53 plasmid and the clone with hygromycin resistance and kanamycin sensitivity was used for further experiments (Δ*crsL*, *JH367*).

Δ*crsL* (*MSMEG_5890*) *strain complementation:* The *MSMEG_5890* coding region including the 150 bp upstream of the start codon was amplified by PCR using the Q5 DNA polymerase (NEB). This fragment was inserted to NotI/PacI digested pTC-mcs plasmid (8) using Gibson assembly. The assembled DNA was then transformed to *E. coli* DH5α competent cells and the sequence verified clone was used for electroporation to the *CrsL* (*MSMEG_5890*) deletion strain (*JH367*). The resulting strain was selected for hygromycin and kanamycin resistance and used in the complementation experiments (Δ*crsL* + *crsL*, *JH410*).

RT-PCR detection of *crsL* mRNA in the *crsL-otsA* operon

To detect which promoters drive the expression of *crsL* mRNA (9) and to detect which mRNA molecules are expressed from the *crsL-otsA* operon, we performed RT-PCR with two primer pairs spanning two pairs of genes: *MSMEG_5889-MSMEG_5890* (*crsL*), and *crsL-MSMEG_5892* (*otsA*). In the primer pair 1, the forward primer was in the *MSMEG_5889* gene and the reverse primer in the c*rsL* gene. In the primer pair 2, the forward primer was in the *crsL* gene, and the reverse primer was in the *otsA* gene. The two amplicons were partially overlapping (see Supplementary Figure 10C). As a template, we used the exponential phase total RNA. As a negative control, reactions without reverse

transcriptase were included. The primers to detect the *MSMEG_5889-MSMEG_5890* (*crsL*) amplicon were: MSMEG_5889_CrsL_F and MSMEG_5889_CrsL_R. The primers to detect the *MSMEG_5890* (*crsL*)-*MSMEG_5892* (*otsA*) amplicon were: CrsL_MSMEG_5892_F and CrsL_MSMEG_5892_R.

Liquid chromatography-tandem mass spectrometry analysis

CarD-FLAG (*LK2539*) and ApeB-FLAG (*LK2767*) *M. smegmatis* strains were grown onto Middlebrook 7H10 (Difco) for 2-3 days at 37 °C supplemented with kanamycin (20 µg/ml). The strains were then inoculated into overnight cultures in Middlebrook 7H9 media (Difco) supplemented with 0.2% glycerol and 0.05% Tween 80 at 37 °C. The overnight cultures were diluted into OD_{600} 0.1 in 100 ml of fresh medium. For exponential phase samples (after 6 hours of growth, OD_{600} ~0.5), anhydrotetracycline (ATc) was added 3 h after inoculation at different concentrations: 1 ng/mL for CarD-FLAG* (optimized) or 10 ng/mL for ApeB-FLAG, and the cultures were then harvested 3 h later. For stationary phase (after 24 hours of growth, OD_{600} ~2.5-3), ATc was added to the CarD-FLAG* culture after 21 h of growth and cells were harvested 3 h later. For the ApeB-FLAG culture, ATc was added 8 h after inoculation and cells were harvested 16 h later. The cells were pelleted and washed in lysis buffer (20 mM Tris–HCl pH 7.9, 150 mM KCl, 1 mM $MgCl_2$) and pelleted again and stored at –70°C. The pellets were resuspended in 3 ml of Lysis buffer supplemented with phenylmethylsulfonyl fluoride (PMSF) and Protease inhibitor cocktail [20 mM Tris–HCl pH 7.9, 150 mM KCl, 1 mM $MgCl_2$, 1 mM dithiothreitol (DTT), 0.5 mM PMSF, Protease Inhibitor Cocktail Set III protease inhibitors (Calbiochem)], sonicated 15 × 10 s with 1 min pauses on ice and centrifuged at 8960 × g for 15 min at 4 °C. Equal amount of cell lysates (2–4 mg of proteins) from the FLAG-tagged strains were incubated for 16–18 h overnight at 4 °C with 25 µl of M2 anti-FLAG resin (Sigma Aldrich). The captured protein complexes with agarose gel beads were washed 4× with 0.5 ml of Lysis buffer. FLAG-tagged proteins were eluted by 60 µl of 3× FLAG Peptide (Sigma F4799) diluted in Tris-buffer saline (TBS: 50 mM Tris-HCl pH 7.5, 150 mM NaCl) to a final concentration of 150 ng/ml. The eluted proteins were digested with trypsin overnight at pH 8.5. The resulting peptides were separated on UltiMate 3000 RSLCnano system (Thermo Fisher Scientific) coupled to Mass Spectrometer Orbitrap Fusion Lumos (Thermo Fisher Scientific). The peptides were trapped and desalted with 2% acetonitrile in 0.1% formic acid at flow rate of 5 µl/min on an Acclaim PepMap100 column (5 µm, 5 mm by 300-µm internal diameter [ID]; Thermo Fisher Scientific). Eluted peptides were separated using an Acclaim PepMap100 analytical column (2 µm, 50-cm by 75-µm ID; Thermo Fisher Scientific). The 125-min elution gradient at a constant flow rate of 300 nl/min was set to 5% phase B (0.1% formic acid in 99.9% acetonitrile) (phase A, 0.1% formic acid) for the first 1 min and then gradient elution by increasing the content of acetonitrile. The orbitrap mass range was set from *m/z* 350 to 2000, in the MS mode, and the instrument acquired fragmentation spectra for ions of *m/z* 100 to 2000. Proteome Discoverer 2.4 (Thermo Fisher Scientific) was used for peptide and protein identification using Sequest and Amanda as search engines and database of sequences of Mycobacterium Smegmatis_ATCC700084 19 (downloaded from Uniprot KB on 20th of December

2019), and common contaminants. The identified proteins in CarD-FLAG, ApeB-FLAG and wild type samples were listed in file and compared to find the differences.

Identification of motifs in CrsL peaks

To search for motifs associated with CrsL peaks, we extracted 500bp sequences centred on peak centres of all CrsL peaks identified by ChIP-seq or for CrsL peaks with high probability values only (−log10 q-value > 40). For motif discovery (Supplementary Figure 8), we used MEME (version 5.5.7) (10) with following parameters:

- Supplementary Figure 8A: "-objfun cd -dna -revcomp -mod zoops -nmotifs 5 -minsites 300 -markov_order 2 -minw 6 -maxw 12 -allw"
- Supplementary Figure 8B: "-objfun cd -dna -revcomp -mod zoops -nmotifs 5 -minsites 150 -markov_order 2 -minw 6 -maxw 12 -allw"

DNA oligonucleotides

Name	Sequence 5′ to 3′	Usage
ApeB-gFLAG_LA_F	CGTTGTAAAACGACGGCCAGTGCCAAGC TTGCGCTGTTCGACCACGAG	Genomic FLAG tagged ApeB cloning
ApeB-gFLAG_LA_R	CTACTTGTCGTCGTCGTCCTTGTAGTCCG CCGGTGACAAGAACGC	
ApeB-gFLAG_HYG_F	GACTACAAGGACGACGACGACAAGTAG GTTAACGAAATCAATCTAAAGTATATAT GAGTAAAC	
ApeB-gFLAG_HYG_R	GGTGACCCTAGAGAAGTTATCCCGGGGC GTC	
ApeB-gFLAG_RA_F	CCGGGATAACTTCTCTAGGGTCACCGGC ATG	
ApeB-gFLAG_RA_R	TTCGAGCTCGGTACCCGGGGATCCTCTA GAGCACGTCACCTACGAGCAG	
CarD-gFLAG_LA_F	TAAAACGACGGCCAGTGCCAAGCTTATG ATTTTTAAGGTCGGAGAC	Genomic FLAG tagged CarD cloning
CarD-gFLAG_LA R	TTTCGTTAACCTACTTGTCGTCGTCGTC	
CarD-gFLAG_HYG_F	CGACAAGTAGGTTAACGAAATCAATCTA AAGTATATATGAGTAAAC	
CarD-gFLAG_HYG_R	CCCGAACAGAGAAGTTATCCCGGGGCGT C	
CarD-gFLAG_RA_F	GGATAACTTCTCTGTTCGGGACGTAAAC G	
CarD-gFLAG_RA_R	ATTCGAGCTCGGTACCCGGGGATCCCCT GAGGCGTCTGGACAG	
ApeB_FLAG_F	ATTCCATATGGCAGCCAGCCCCCATAGC TTG	FLAG tagged ApeB cloning
ApeB_FLAG_R	CGTAAGCTTCTACTTGTCGTCGTCGTCCT TGTAGTCCGCCGGTGACAAGAACGCCTG	
CrsL_FLAG_F	GCGCATATGCCTGCGAAAACTGATCCC	FLAG tagged CrsL cloning
CrsL_FLAG_R	CGCAAGCTTTTACTTGTCGTCGTCGTCCT TGTAGTCGACCTCGGTCTTCGAGGGCC	
*carD*_sgRNA_F	GGGAACGTGTGATCTGGGCCCGTG	

*carD*_sgRNA_R	AAACCACGGGCCCAGATCACACGT	*carD* knockdown_CRISPR cloning
*crsL*_sgRNA_F	GGGAGGGATCAGTTTTCGCAGGCA	*crsL* knockdown_CRISPR cloning
*crsL*_sgRNA_R	AAACTGCCTGCGAAAACTGATCCC	
CrsL-His_F	GCCGAGCATATGCCTGCGAAAACTGATC CCGC	Cloning of the expression vector for CrsL
CrsL-His_R	GGCTGCTCGAGGACCTCGGTCTTCGAGG GCCC	
carD_F	ACACCGTCGTCTATCCACAC	RT-qPCR
carD_R	TTGTCCGCTGGTACTCGAA	RT-qPCR
crsL_F	ATGCCTGCGAAAACTGATCC	RT-qPCR
crsL_R	GACAGGAACATGCGGCATTC	RT-qPCR
sigA_F	CCAAGGGCTACAAGTTCTCG	RT-qPCR
sigA_R	CTTGTTGATCACCTCGACCA	RT-qPCR
PLAt_spike_F	TTACTGCAGCTGAGGTCACA	RT-qPCR
PLAt_spike_R	AGCTCACACTCTGTCCAGTC	RT-qPCR
CrsL_GA_LA_fwd	GCCAGTGCCAAGCTTGCATGCGCCCAGT TCAGGGCCGC	*crsL* deletion strain cloning
CrsL_GA_LA_rev	CTTGTGTCATGAGAACAACTGTAGGCGA CCGGG	
CrsL_GA_hyg_fwd	AGTTGTTCTCATGACACAAGAATCCCTGT TACTTCTCGACCGTATTGATTCGGATGAT TCCTACGC	
CrsL_GA_hyg_rev	CCGGAGACATTCAGGCGCCGGGGGCGGT	
CrsL_GA_RA_fwd	CGGCGCCTGAATGTCTCCGGAGAGTGGC	
CrsL_GA_RA_rev	ATTCGAGCTCGGTACCCGGGTCTGCATG AACAGCTCGAC	
Crsl_prom+coding1_fwd	TACGTCGACATCGATTTAATTAACTAGAC CTCGGTCTTCGAGGG	*crsL* deletion strain complementation cloning
Crsl_prom+coding1_rev	CGACCGAGCGCAACGCGTGCGGCCGCCA GTGCATCGGCCTGGCC	
DnaK_F	ATTCCATATGGCTCGTGCGGTCGGTATCG AC	Cloning of the expression vector for DnaK
DnaK_pET22b_R	CCGAAGCTTCTTGTTCTCCTTGCCAGCGT C	
MSMEG_5889_CrsL_F	GGCAACAACACGGGTCAG	RT-PCR to detect the mRNA molecules expressed from the *crsL-otsA* operon
MSMEG_5889_CrsL_R	GACAGGAACATGCGGCATTC	
CrsL_MSMEG_5892_F	GACGTGGAACCGCTTGCC	
CrsL_MSMEG_5892_R	CTCCAGATCGACCGGTAGC	

PLAT mRNA spike-in sequence (718 bp):

GGGAGAGCGGCCGCATGGGCAAGAGTTACACAGCGTGGAGGACCAACTCCCAGGCACTCGGCCTG
GGCAGACACAATTATTGTCGGAATCCAGATGGTGATGCCAGACCTTGGTGCCATGTGATGAAGGAC
CGAAAGCTGACGTGGGAATACTGTGACATGTCCCCATGCTCCACCTGTGGCCTGAGGCAGTACAAA
CGGCCTCAGTTTAGAATTAAAGGAGGACTCTACACAGACATCACCTCACACCCTTGGCAGGCTGCC

ATCTTTGTCAAGAACAAGAGGTCTCCTGGAGAGAGATTCCTTTGTGGAGGGGTGCTGATCAGTTCC
TGCTGGGTGCTGTCAGCTGCCCACTGCTTTCTAGAGAGGTTCCCCCCCAATCATCTTAAAGTGGTCT
TGGGCAGAACATACAGGGTGGTCCCCGGAGAGGAAGAACAGACATTTGAGATTGAAAAATACATA
GTCCATGAGGAATTTGATGACGATACTTATGACAACGACATCGCATTACTGCAGCTGAGGTCACAG
TCCAAGCAATGTGCCCAAGAGAGCAGCTCTGTTGGCACTGCCTGCCTCCCTGACCCCAACCTGCAG
CTCCCTGACTGGACAGAGTGTGAGCTTTCTGGCTACGGCAAGCATGAGGCATCGTCTCCATTCTTCT
CTGCGGCCGCAATCTTTCTAGAAGATCTCCTACAATATTCTCAGCTGCCATGGAAAAT

Bacterial strains

Strain number	Organism	Usage
LK2598 (WT, wild type)	***Mycobacterium smegmatis*** **mc^2 155**	western blots, IP, RNA-seq, ChIP-seq
LK2846 (ApeB-gFLAG)	*Mycobacterium smegmatis*	western blots, IP
LK2899 (CarD-gFLAG)	*Mycobacterium smegmatis*	western blots, IP
LK2767 (ApeB-FLAG)	*Mycobacterium smegmatis*	western blots, IP, mass spectrometry
LK2539 (CarD-FLAG)	*Mycobacterium smegmatis*	ChIP-seq, western blots, northern blots, mass spectrometry
LK3051 (CrsL-FLAG)	*Mycobacterium smegmatis*	western blots, IP, ChIP-seq
LK2261 (*nc*WT)	*Mycobacterium smegmatis*	western blots, RT-qPCR, northern blots, RNA-seq
LK2758 (*sigA* knockdown)	*Mycobacterium smegmatis*	western blots, RT-qPCR, northern blots
LK3301 (*carD* knockdown)	*Mycobacterium smegmatis*	western blots, RT-qPCR, northern blots, RNA-seq
LK3302 (*crsL* knockdown)	*Mycobacterium smegmatis*	western blots, RT-qPCR, northern blots, RNA-seq
JH7 (RNAP-FLAG)	*Mycobacterium smegmatis*	IP
JH151 (RNAP-FLAG + *crsL* knockdown)	*Mycobacterium smegmatis*	IP
JH171 (RNAP-FLAG + *nc*WT)	*Mycobacterium smegmatis*	IP
JH367 (Crsl deletion strain)	*Mycobacterium smegmatis*	Growth curves, RT-qPCR
JH410 (complemented CrsL deletion strain)	*Mycobacterium smegmatis*	Growth curves, RT-qPCR
LK3209 (CarD-NT)	*E. coli* DE3	CarD purification strain
LK3499 (CrsL-His)	*E. coli* DE3	CrsL purification strain
JH185 (DnaK-His)	*E. coli* DE3	DnaK purification strain

Antibodies

Target	Clone	Source
RNA polymerase β	8RB13	BioLegend Cat. No. 663903
σ^{70}	2G10	BioLegend Cat. No. 663207
CarD		home-made
CrsL		home-made
RbpA		home-made
FLAG peptide		Merck Cat. No. F3165
GroEL	5177	Santa Cruz Biotechnology sc-58170
HRP		Sigma-Aldrich Cat. No. A9044

SUPPLEMENTARY REFERENCES

1. Vaňková Hausnerová, V., Kumar, D., Shoman, M., Schwarz, M., Modrák, M., Jirát Matějčková, J., Neva, S., Havelková, J., Šiková, M., Pospíšilová, D. *et al.* (2024) HelD is a Global Transcription Factor Enhancing Gene Expression in Rapidly Growing Mycobacteria. *bioRxiv*, 2024.2011.2027.625628.
2. Choudhary, E., Thakur, P., Pareek, M. and Agarwal, N. (2015) Gene silencing by CRISPR interference in mycobacteria. *Nat Commun*, **6**, 6267.
3. Kouba, T., Koval, T., Sudzinová, P., Pospíšil, J., Brezovská, B., Hnilicová, J., Šanderová, H., Janoušková, M., Šiková, M. and Halada, P. (2020) Mycobacterial HelD is a nucleic acids-clearing factor for RNA polymerase. *Nature communications*, **11**, 1-13.
4. Rock, J.M., Hopkins, F.F., Chavez, A., Diallo, M., Chase, M.R., Gerrick, E.R., Pritchard, J.R., Church, G.M., Rubin, E.J. and Sassetti, C.M. (2017) Programmable transcriptional repression in mycobacteria using an orthogonal CRISPR interference platform. *Nature microbiology*, **2**, 1-9.
5. Šiková, M., Janoušková, M., Ramaniuk, O., Páleníková, P., Pospíšil, J., Bartl, P., Suder, A., Pajer, P., Kubičková, P. and Pavliš, O. (2019) Ms1 RNA increases the amount of RNA polymerase in Mycobacterium smegmatis. *Molecular Microbiology*, **111**, 354-372.
6. Huff, J., Czyz, A., Landick, R. and Niederweis, M. (2010) Taking phage integration to the next level as a genetic tool for mycobacteria. *Gene*, **468**, 8-19.
7. van Kessel, J.C. and Hatfull, G.F. (2007) Recombineering in Mycobacterium tuberculosis. *Nat Methods*, **4**, 147-152.
8. Klotzsche, M., Ehrt, S. and Schnappinger, D. (2009) Improved tetracycline repressors for gene silencing in mycobacteria. *Nucleic Acids Res*, **37**, 1778-1788.
9. Martini, M.C., Zhou, Y., Sun, H. and Shell, S.S. (2019) Defining the Transcriptional and Post-transcriptional Landscapes of. *Front Microbiol*, **10**, 591.
10. Bailey, T.L. and Elkan, C. (1994) Fitting a mixture model by expectation maximization to discover motifs in biopolymers. *Proc Int Conf Intell Syst Mol Biol*, **2**, 28-36.

Supplementary Figure 1.

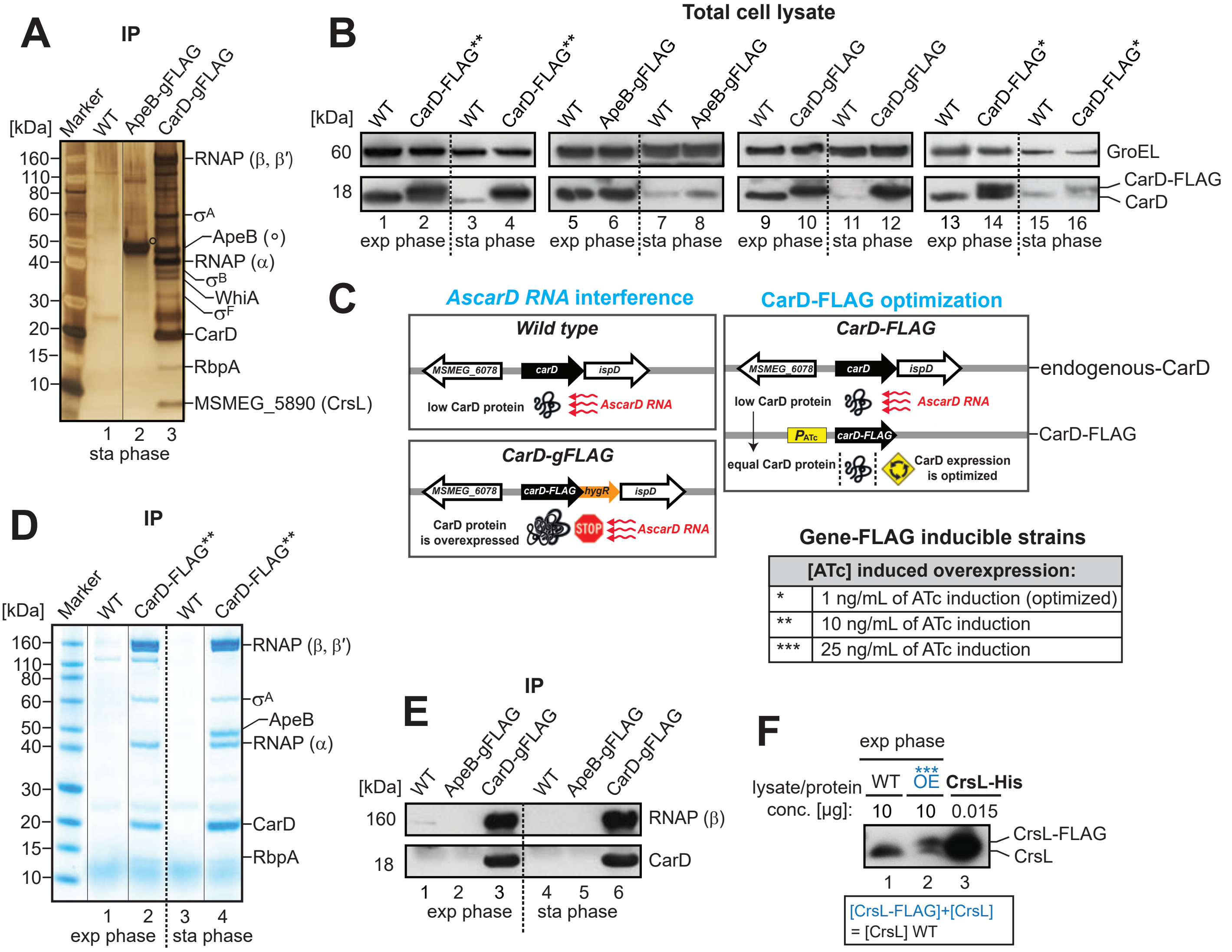

Supplementary Figure 1. A. Proteins co-immunoprecipitated with ApeB-gFLAG and CarD-gFLAG from stationary phase were resolved by SDS PAGE and visualized by silver-staining. **B.** Western blot of total cell lysates from *M. smegmatis* wild type and CrsL-FLAG** (10 ng/mL ATc), the optimized CarD-FLAG* (1 ng/mL ATc), CarD-gFLAG and ApeB-gFLAG strains from exponential and stationary phase. The level of CarD was detected by anti-CarD antibody. GroEL was used as loading control and detected with anti-GroEL antibody. Note that the presence of a FLAG tag slows down the migration of the tagged protein compared to the non-tagged protein. **C.** Schematic representation of *AscarD* RNA interference and CarD level optimization in CarD-FLAG strain. **D.** Proteins co-immunoprecipitated with CarD-FLAG** (10 ng/mL ATc) from exponential and stationary phases were resolved by SDS PAGE and visualized by Coomassie. **E.** Proteins co-immunoprecipitated with ApeB-gFLAG and CarD-gFLAG from stationary phase cultures were analyzed by western blotting using anti-RNAP (β) and anti-CarD antibodies. **F.** The levels of endogenous CrsL and the overexpressed CrsL-FLAG (25 ng/mL ATc) from 10 μg of cell lysates were compared to a 15 ng of *in vitro* purified CrsL-His protein and detected by western blotting with anti-CrsL antibody. Asterisks indicate the different ATc concentrations (ng/mL) used in the inducible FLAG-tagged strains.

Supplementary Figure 2.

A

CrsL AlphaFold predicted structure

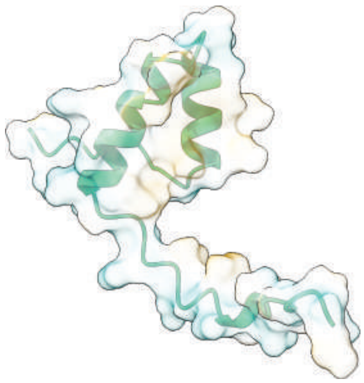

B

SSP scores computed for Cα, Cβ, N, H

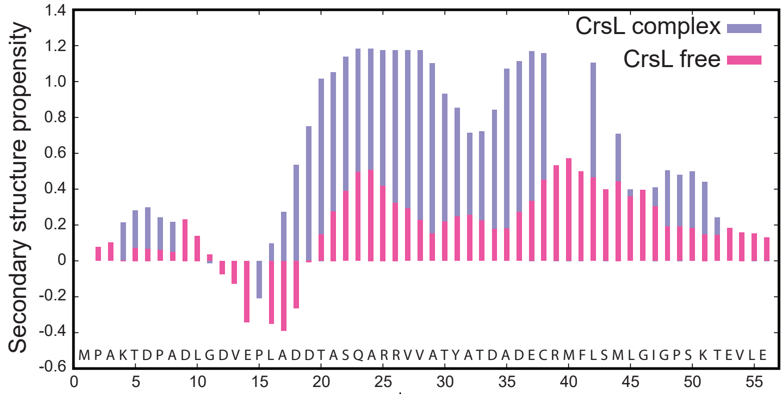

ncSP scores computed for Cα, Cβ, N, H

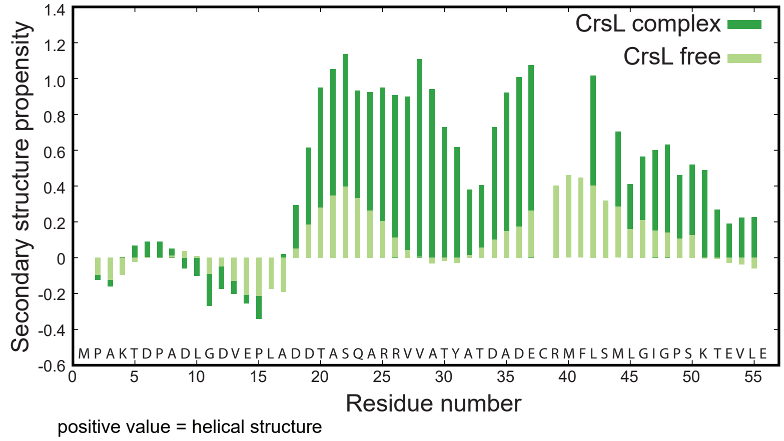

positive value = helical structure
negative value = beta sheets

C

Isothermal titration calorimetry (ITC)

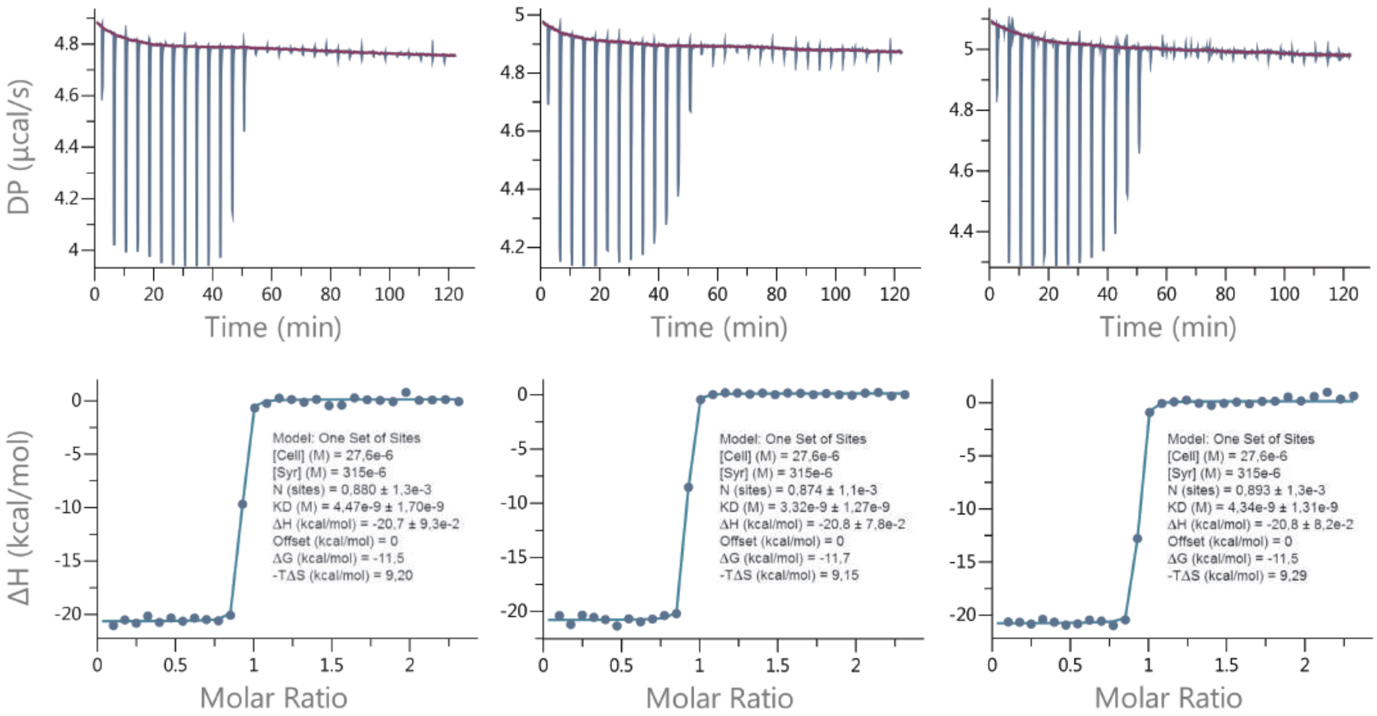

Supplementary Figure 2. A. Predicted structure of CrsL calculated by AlphaFold. The accessible surface area is colored based on hydrophobicity; surfaces are colored from hydrophilic (dark cyan) to hydrophobic (gold). **B.** SSP and ncSP scores calculated for both free and bound CrsL using all available chemical shifts of Cα, Cβ, N and H. Positive and negative values correspond to helical and extended conformations, respectively. **C.** Three independent ITC experiments, with thermogram at the top and corresponding titration curves at the bottom, evaluating CrsL-CarD interaction. The concentration of the CarD (in the cell) was 27.6 μM, the concentration of the CrsL in the syringe was 315 μM.

Supplementary Figure 3.

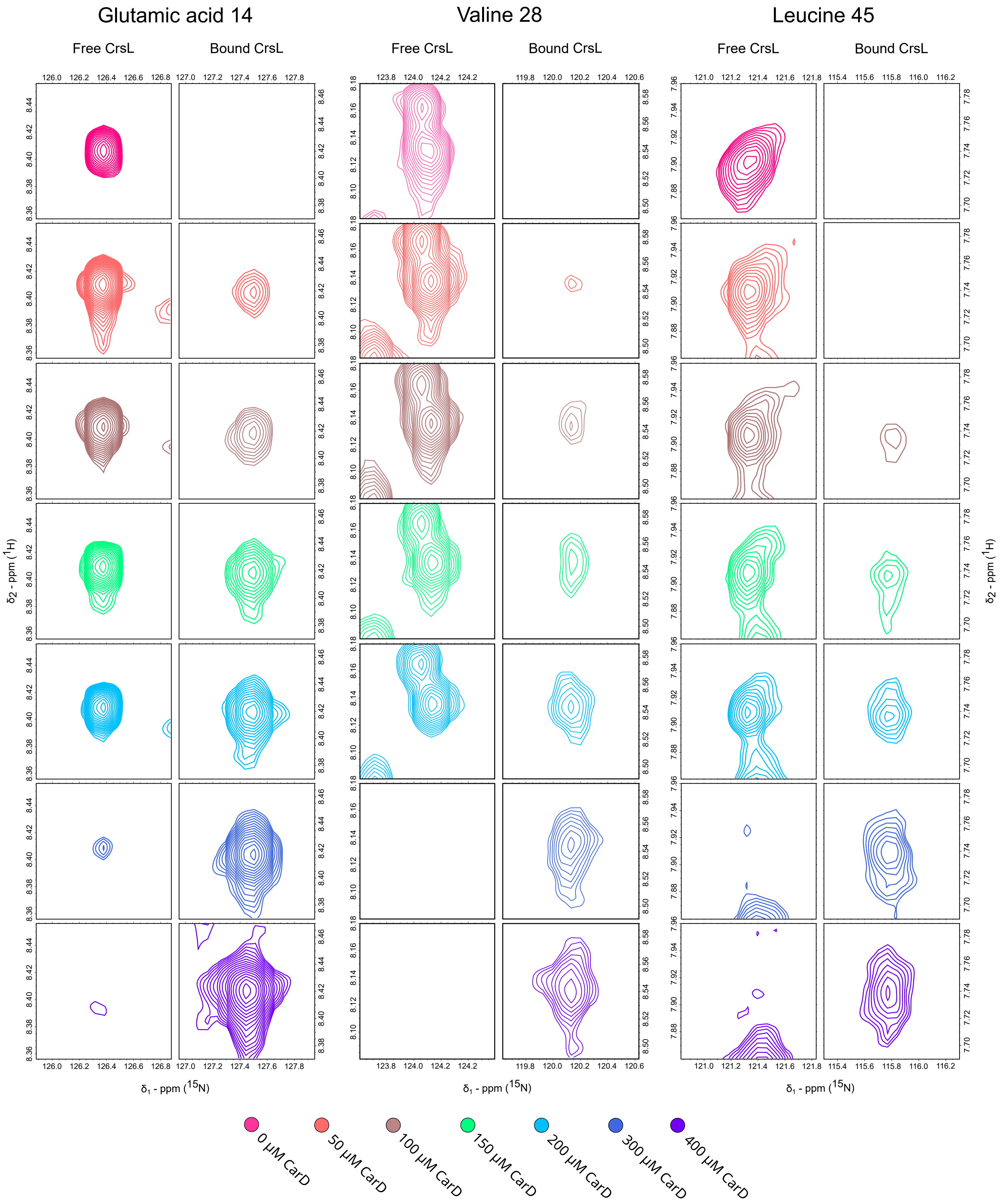

Supplementary Figure 3. Details of peak intensity changes in 2D ^{1}H–^{15}N HSQC spectra of 200 μM ^{15}N-labelled CrsL during titration with unlabeled CarD. Colors corresponding to individual concentrations of CarD are indicated below the spectra. Examples of well-resolved peaks of free and bound CrsL are displayed in boxes.

Supplementary Figure 4.

A

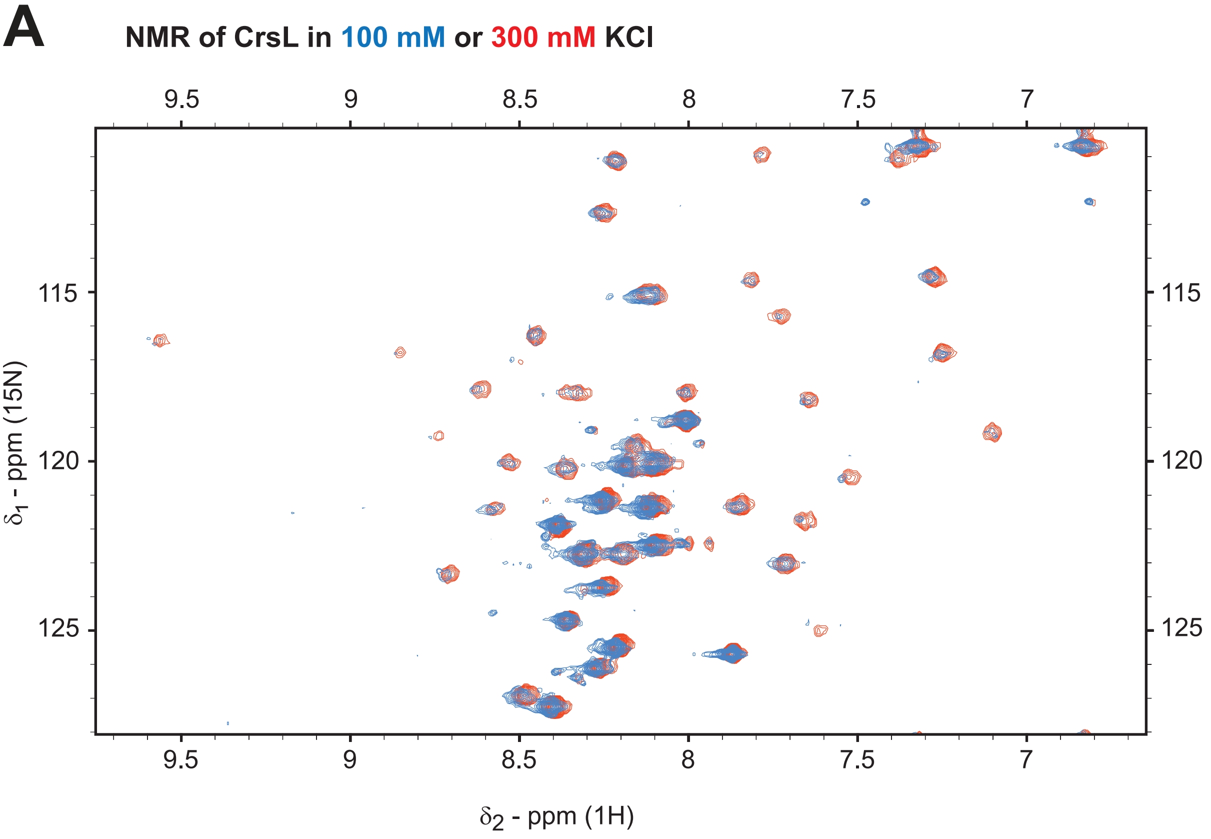

B

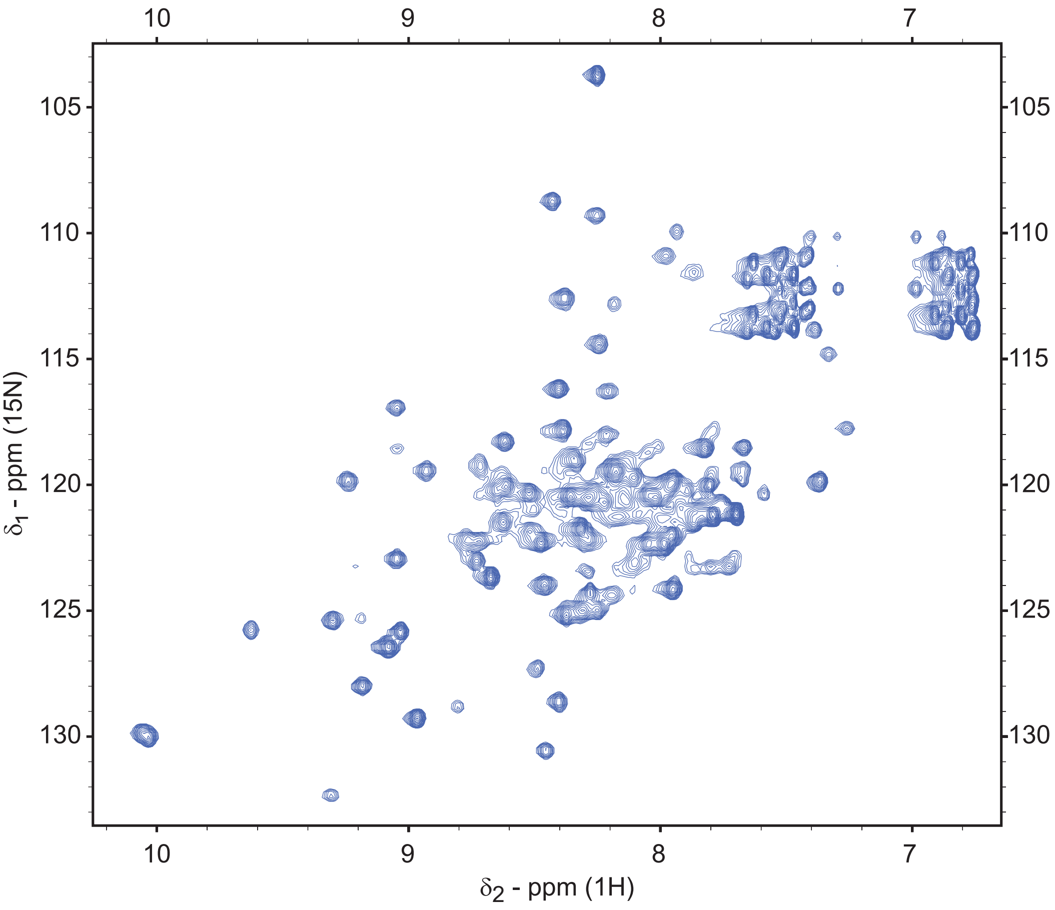

Supplementary Figure 4. A. Impact of increasing of the salt concentration (blue 100mM, orange 300mM sodium chloride) on the signal broadening in 2D ^{1}H–^{15}N HSQC spectrum of ^{15}N-labeled CrsL in complex with CarD. **B.** 2D ^{1}H–^{15}N TROSY spectrum of 1mM free [^{13}C, ^{15}N]-CarD acquired with 256 scans.

Supplementary Figure 5.

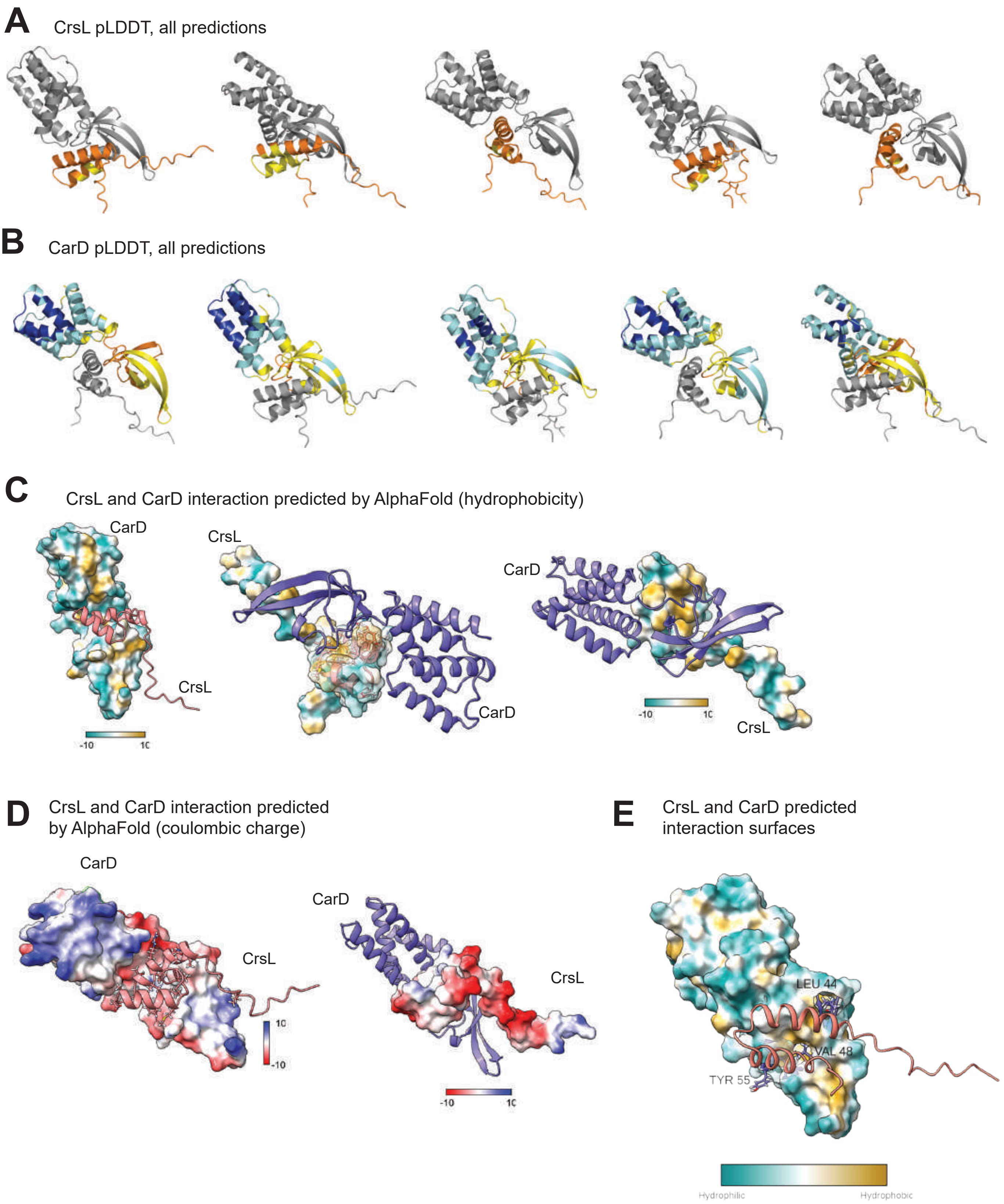

Supplementary Figure 5. A. Five ranked structures of the CrsL-CarD complex predicted by AlphaFold Multimer. CarD is colored grey and CrsL is colored based on predicted local distance difference test (pLDDT). pLDDT is a per-residue measure of local confidence ranging between 0 and 100, where values of yellow (<70) and orange show (<50) low confidence and light (<90) and dark blue (>90) show high confidence. **B.** Five best structures of CrsL-CarD complex predicted by AlphaFold Multimer with CrsL colored grey and CarD colored based on pLDDT. **C.** The AlphaFold Multimer prediction of the CrsL-CarD complex color-coded according to the hydrophobicity; the colors range from dark cyan (hydrophylic) to gold (hydrophobic). Hydrophobicity potential was calculated using the ChimeraX mlp command. **D.** The AlphaFold Multimer prediction of the CrsL-CarD complex color-coded according to the coulombic electrostatic potential. The colors range from blue (positive charge) to red (negative charge). Electrostatic potential was calculated using the ChimeraX coulombic command. **E.** The AlphaFold Multimer prediction of the CrsL-CarD complex colored by hydrophobicity. Important residues with sidechains (Tyr55, Val48 and Leu44) are displayed.

Supplementary Figure 6.

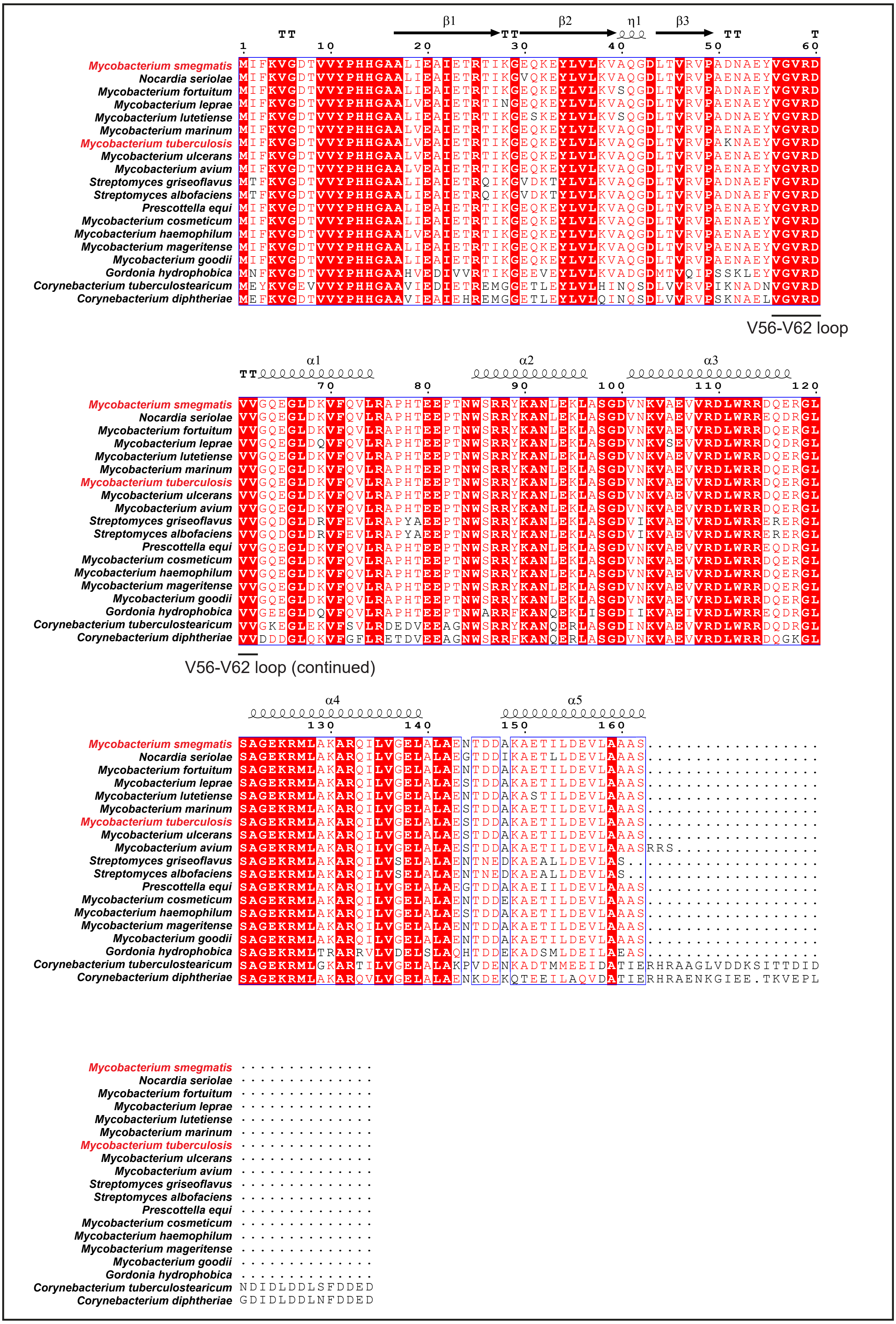

Supplementary Figure 6. Multiple sequence alignment of 19 CarD homologs with visualized secondary structure at the top, based on solved structure of *M. tuberculosis* CarD (PDB 4ILU). CarD is generally well conserved across different species of actinobacteria. CarD-CrsL interaction site including the interdomain loop (V56-V62) is particularly well conserved.

Supplementary Figure 7.

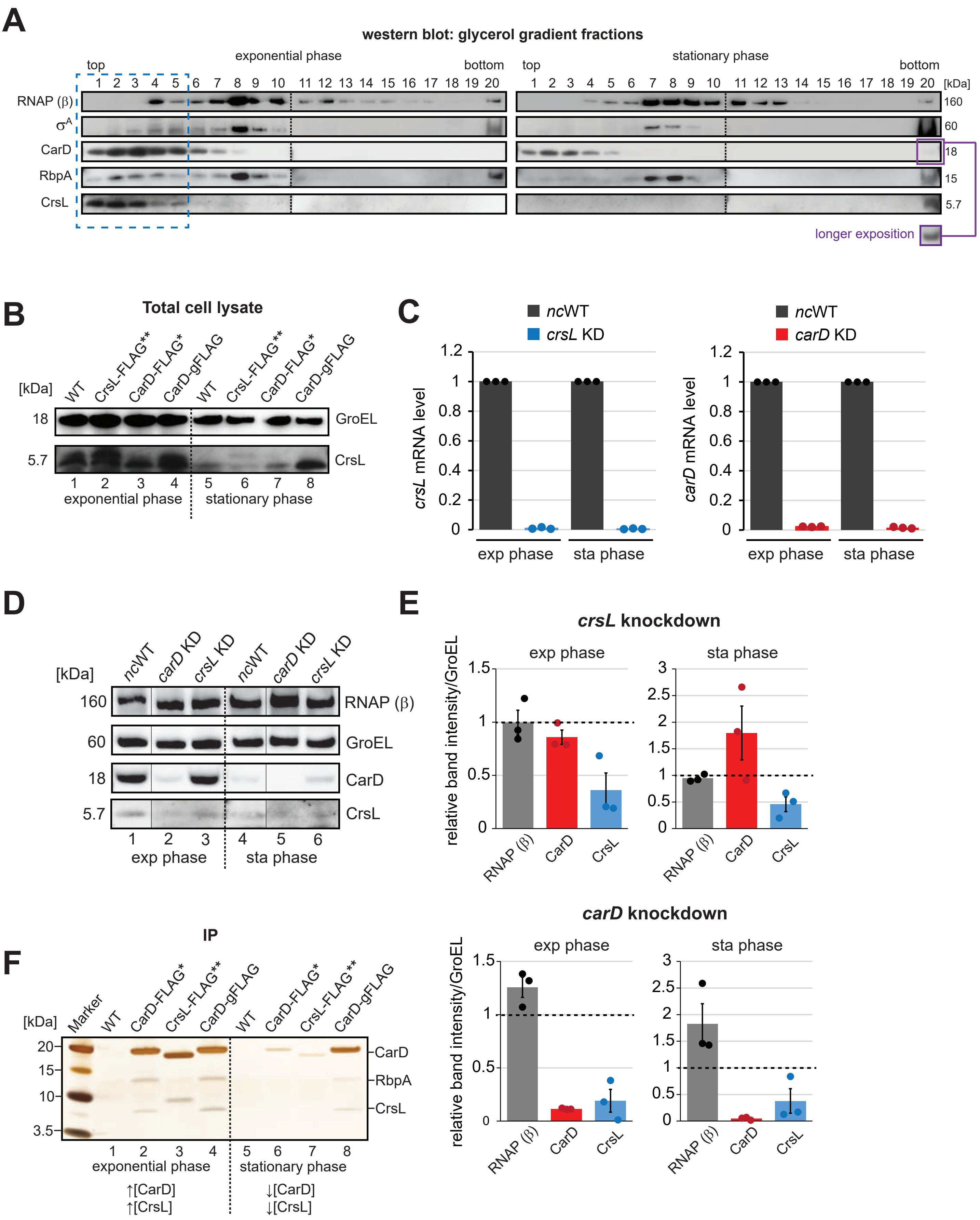

Supplementary Figure 7. A. Total protein lysates from *M. smegmatis* exponential and stationary phase cells were separated by glycerol gradient ultracentrifugation. The amounts of RNAP, σ^A, CarD, RbpA and CrsL in individual fractions were detected by western blotting using anti-RNAP (β), anti-σ^{70}, anti-CarD, anti-RbpA and anti-CrsL antibodies, respectively. **B.** Western blot of total cell lysates from *M. smegmatis* wild type and CrsL-FLAG** (10 ng/mL ATc), the optimized CarD-FLAG* (1 ng/mL ATc) and CarD-gFLAG strains from exponential and stationary phase. The level of CrsL was detected by anti-CrsL antibody. GroEL was used as loading control and detected with anti-GroEL antibody. **C.** Depletion of *crsL* and *carD* using CRISPR. The mRNA levels of depleted *crsL* and *carD* genes in *crsL* knockdown and *carD* knockdown strains, respectively, in exponential and stationary phase were measured by RT-qPCR with gene specific primers. The data was normalized to spike-in control and negative control (*nc*WT) was set as 1. Three-four biological replicates were used, and error bars represent SEM. **D.** The protein levels of RNAP (β), CarD and CrsL upon depletion of *crsL* and *carD* detected by western blotting using anti-RNAP (β), anti-CarD and anti-CrsL antibodies. GroEL level was used as loading control and *nc*WT was used as a negative control. **E.** The quantified western blot bands of protein levels of RNAP (β), CarD and CrsL upon depletion of *crsL* and *carD*, from biological triplicates. The data was normalized to GroEL level, and the negative control (*nc*WT) was set as 1. Error bars represent SEM. **F.** Immunoprecipitated proteins with the optimized CarD-FLAG* (1 ng/mL ATc), CrsL-FLAG** (10 ng/mL ATc) and CarD-gFLAG strains from exponential and stationary phase visualized by silver stained SDS-PAGE. Note that the presence of a FLAG tag slows down the migration of the tagged protein compared to the non-tagged protein.

Supplementary Figure 8.

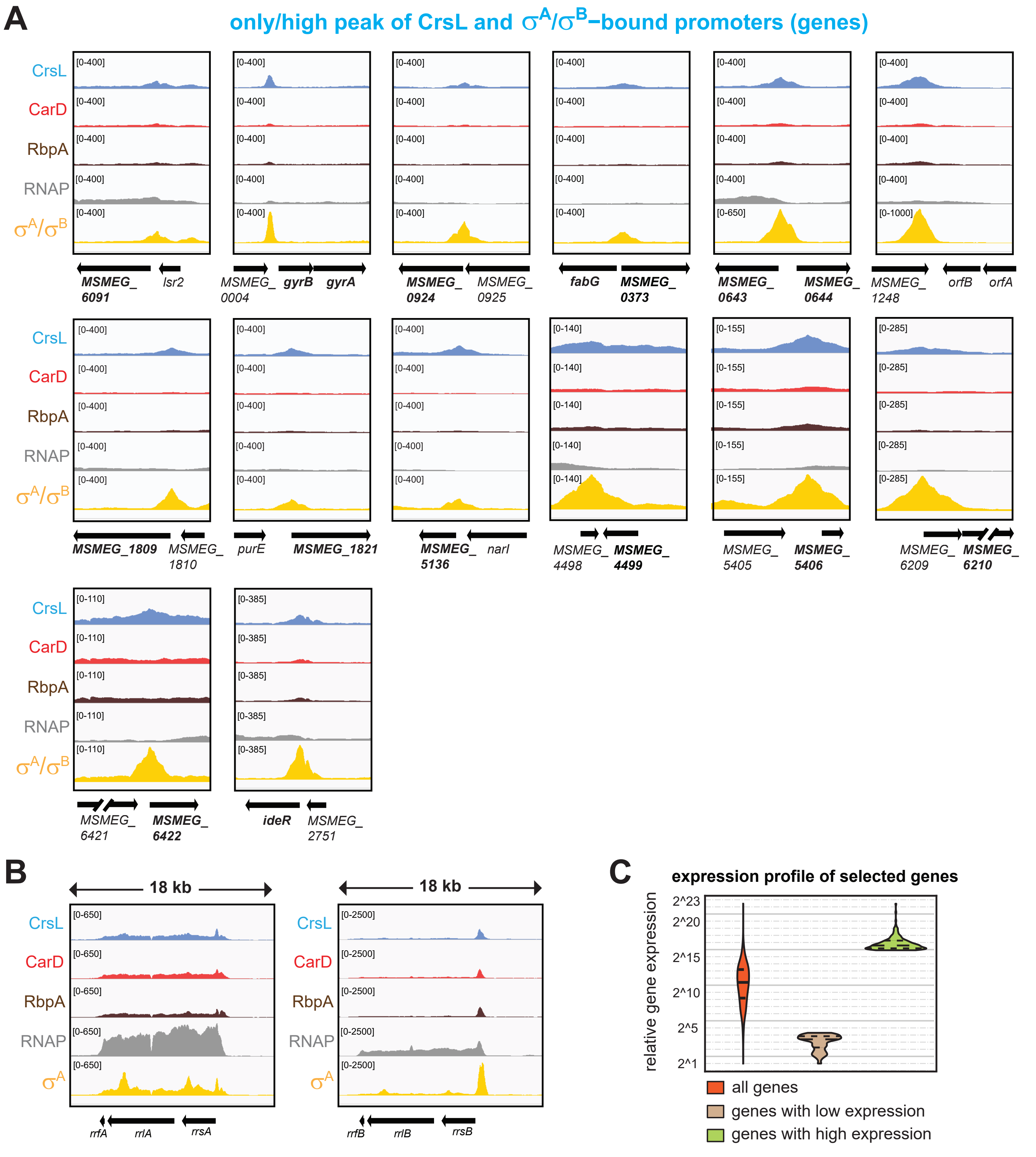

Supplementary Figure 8. A. Genes with CrsL and σ^A/σ^B peaks as detected by ChIP-seq. **B.** ChIP-seq peaks detected on the promoters of rRNAs. **C.** Expression profile of all genes, genes with no/low expression and highly expressed genes in exponential and stationary phase in *M. smegmatis*. The plot was generated from the data obtained from Šiková et al. (1).

Supplementary Figure 9.

A **all CrsL peaks**

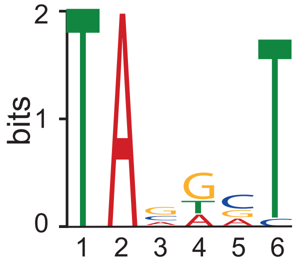

B **CrsL peaks with q-value > 40**

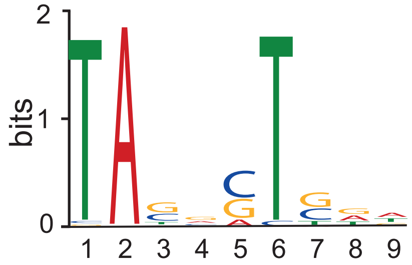

C **Model of Transcription initiation complex with σ^A, CarD, RbpA and CrsL**

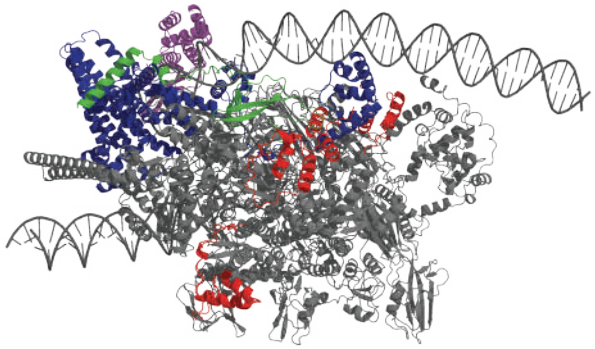

D **Model of Transcription initiation complex with σ^A (now in dark grey), CarD, RbpA and CrsL**

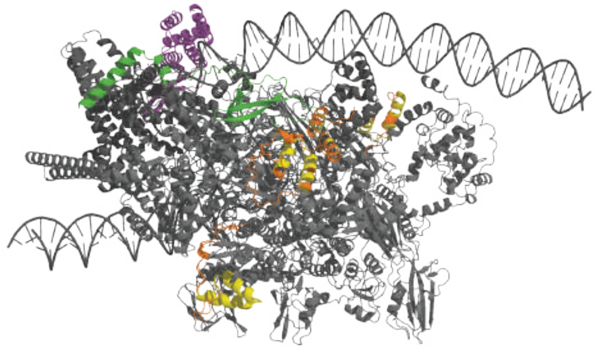

Supplementary Figure 9. A. DNA motifs enriched at all CrsL peaks identified by ChIP-seq, or **B.** at CrsL peaks with high probability values only ($-\log_{10}$ q-value > 40). CrsL is mainly bound to DNA sequences partially resembling −10 promoter elements. **C.** AlphaFold3 model of mycobacterial RNA polymerase (grey) holoenzyme with DNA (grey), σ^A (blue), RbpA (green), CarD (purple) and CrsL (red, multiple sites). 5 best predictions of CrsL are superimposed. AlphaFold fails to predict consistent binding site for CrsL, instead it positions it on 3 different spots on the surface of β' subunit. Interestingly, it fails at accounting the interaction between CrsL and CarD. **D.** The same superimposition with CrsL colored by local confidence score (pLDDT), with σ^A colored grey for better visibility. AlphaFold3 score is low, illustrating a challenge of predicting a novel interaction between mycobacterial holoenzyme CarD and CrsL.

Supplementary Figure 10.

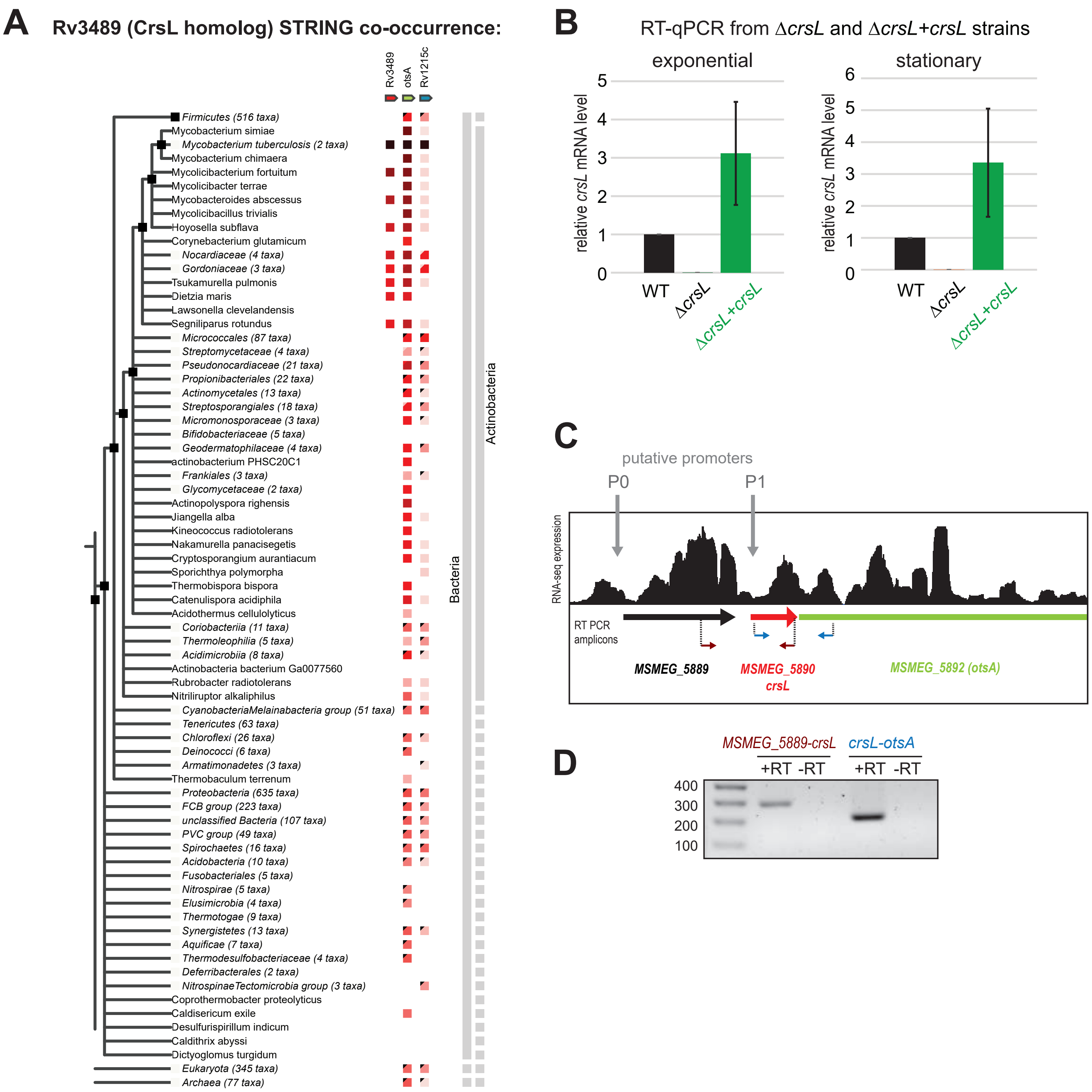

Supplementary Figure 10. A. The co-occurrence of the CrsL homolog (*Rv3489*) in bacteria from the STRING database (2). **B.** RT-qPCR data demonstrating the relative level of *crsL* mRNA in exponential and stationary phase in WT, Δ*crsL* and Δ*crsL* + *crsL* strains. The data was normalized to spike-in control and the WT strain was set as 1. **C.** *crsL-otsA* operon in *M. smegmatis* and *MSMEG_5889* gene upstream of this operon. The promoters were annotated based on the transcription start sites reported by Martini *et al*., 2019 (3). The RNA-seq expression profile is from the exponential phase wild-type *M. smegmatis*, and the image was adapted from https://msmegseq.elixir-czech.cz/. **D.** RT-PCR confirmed the presence of *crsL-otsA* mRNA, but also *MSMEG_5889-crsL* mRNA (for primer positions, see the scheme below the genomic region shown in C). '-RT' shows the RT-PCR reaction with no reverse transcriptase added and represents a negative control for possible contamination of genomic DNA in the RNA sample.
